# Identifying Hmga2 preserving visual function by promoting a shift of Müller glia cell fate in mice with acute retinal injury

**DOI:** 10.1186/s13287-024-03657-9

**Published:** 2024-02-27

**Authors:** Zhiyuan Yin, Lingling Ge, Zhe Cha, Hui Gao, Luodan A, Yuxiao Zeng, Xiaona Huang, Xuan Cheng, Kai Yao, Zui Tao, Haiwei Xu

**Affiliations:** 1 Southwest Eye Hospital, Southwest Hospital, Third Military Medical University (Army Medical University), Chongqing, 400038 China; 2 Key Lab of Visual Damage and Regeneration and Restoration of Chongqing, Southwest Eye Hospital, Southwest Hospital, Chongqing, 400038 China; 3 Department of Physiology, College of Basic Medical Sciences, Third Military Medical University (Army Medical University), Chongqing, 400038 China; 4 Institute of Visual Neuroscience and Stem Cell Engineering, College of Life Sciences and Health, Wuhan University of Science and Technology, Wuhan, 430065 China

**Keywords:** Hmga2, Müller glia, Gliosis, Reprogramming, Epigenetic, Proliferation, Bulk-RNA seq, scRNA seq, Visual restoration

## Abstract

**Background:**

Unlike in lower vertebrates, Müller glia (MG) in adult mammalian retinas lack the ability to reprogram into neurons after retinal injury or degeneration and exhibit reactive gliosis instead. Whether a transition in MG cell fate from gliosis to reprogramming would help preserve photoreceptors is still under exploration.

**Methods:**

A mouse model of retinitis pigmentosa (RP) was established using MG cell lineage tracing mice by intraperitoneal injection of sodium iodate (SI). The critical time point for the fate determination of MG gliosis was determined through immunohistochemical staining methods. Then, bulk-RNA and single-cell RNA seq techniques were used to elucidate the changes in RNA transcription of the retina and MG at that time point, and new genes that may determine the fate transition of MG were screened. Finally, the selected gene was specifically overexpressed in MG cells through adeno-associated viruses (AAV) in the mouse RP model. Bulk-RNA seq technique, immunohistochemical staining methods, and visual function testing were used to elucidate and validate the mechanism of new genes function on MG cell fate transition and retinal function.

**Results:**

Here, we found the critical time point for MG gliosis fate determination was 3 days post SI injection. Hmga2 was screened out as a candidate regulator for the cell fate transition of MG. After retinal injury caused by SI, the Hmga2 protein is temporarily and lowly expressed in MG cells. Overexpression of Hmga2 in MG down-regulated glial cell related genes and up-regulated photoreceptor related genes. Besides, overexpressing Hmga2 exclusively to MG reduced MG gliosis, made MG obtain cone’s marker, and retained visual function in mice with acute retinal injury.

**Conclusion:**

Our results suggested the unique reprogramming properties of Hmga2 in regulating the fate transition of MG and neuroprotective effects on the retina with acute injury. This work uncovers the reprogramming ability of epigenetic factors in MG.

**Supplementary Information:**

The online version contains supplementary material available at 10.1186/s13287-024-03657-9.

## Background

Retinal degenerative diseases, such as age-related macular degeneration (AMD) and retinitis pigmentosa (RP), are refractory and irreversible eye diseases that caused by the death of photoreceptors and/or retinal pigment epithelium (RPE) cells [1]. In adult mammals, the retina lacks the ability to regenerate itself after injury or degeneration [2]. As a result, when RPEs, photoreceptors, or other neurons are severely damaged, there are no viable treatments to restore visual function in mammals. Stem cell transplantation has achieved several successes in experimental and clinical research [3]. However, the source of donor cells, safety, and efficacy are constant bottleneck issues. It is becoming more and more appealing to activate endogenous stem cells, mainly Müller glia (MG), to repair or regenerate the diseased or injured retina [4].

When retinal injury occurred in zebrafish, MG could be driven to de-differentiate, proliferate as progenitor-like cells, create offspring cells, differentiate into functional neurons, and finally restore visual function [5]. Mammalian MG, on the other hand, lack the ability to differentiate into stem cells or retinal progenitor cells (RPCs). At the initial stage of retinal injury, only a small number of MG could be stimulated to proliferate and progress to neurogenesis. Retinal stress is typically caused by injury or degeneration, which aids in MG enlargement and increased stiffness [6–8]. At the late stages of retinal injury, MG suffered gliosis fate as opposed to reprogramming fate, nevertheless [9]. MG gliosis is characterized by glial hypertrophy, the up-regulation of intermediate filament proteins, particularly glial fibrillary acidic protein (GFAP) and Vimentin, proliferation and migration [10, 11]. Retinal injury finally triggers MG to establish a glial scar to fill retinal breaks and to replace degenerated neurons, prevents neuronal cell migration, axonal regrowth, and limits endogenous regeneration [12–14].

Many efforts have been made in the past decade to achieve MG reprogramming in mammal. Overexpression or knockout of certain genes, especially the transcription factors involved in retinal development, such as Ascl1, Otx2, Crx, Nrl, Pou4f2, Atoch7, Islet, NFI, Ikzf1, Ikzf4, Ptbp1 and NeuroD1, was widely adopted to achieve MG reprogramming in mice [15–22]. Although reprogrammed MG changed their cell fate from gliosis to reprogramming, they did not improve visual function significantly [23]. When MG reprogrammed, many epigenetic traits of the MG would be remodeled. Changing epigenetic characteristics of MG, such as DNA methylation, DNA acetylation, histone modification or chromatin compaction, could remove the constraints on the expression of differentiated traits and create environments that maintain stemness, even restore vision [24, 25]. Hence, we hypothesized that direct epigenetic remodeling is an attractive approach to MG reprogramming achievement.

In this study, the critical time point for MG gliosis occurrence was determined by analyzing dynamical responses of retina and MG to acute retinal injury. At this time point, High mobility group A2 (Hmga2), an epigenetic regulator, was screened out to be potentially involved in switching MG cell fate. Adeno-associated virus (AAV) was used to deliver Hmga2 into adult mouse MG for overexpression. Overexpressed Hmga2 reduced MG gliosis, made MG obtain cone’s marker and protected visual function after acute retinal injury.

## Material and methods

### Animals

The manuscript adheres to the ARRIVE guidelines for the reporting of animal experiments. Glast-Cre^ER^ (Tg (Slc1a3-Cre/ERT) 1Nat/J) transgenic mice (the Jackson Laboratory, stock no. 012586) were crossed with Cre-inducible CAG-LSL-tdTomato reporter B6/JNju-H11^em1Cin(CAG−LoxP−ZsGreen−Stop−LoxP−tdTomato)^/Nju, which were purchased from JiCuiYaoKang Biology Company (Nanjing, China) to establish the MG lineage mice. C57BL/6J (C57) mice were purchased from the Experimental Animal Center of the Army Military Medical University. Mice were maintained in the Animals Center of the Army Medical University with free access to standard food and water at room temperature 25 °C under a 12/12 h light/dark cycle. All experimental procedures were approved by the Faculty Committee on the Use of Live Animals in Teaching and Research (Third Military Medical University). All animal experiments were conducted following the Association for Research in Vision and Ophthalmology Statement.

### Tamoxifen and sodium iodate injection

To induce the expression of tdTomato, tamoxifen (Sigma, T5648) (100 mg/kg body weight) in corn oil was injected intraperitoneally. To create a mouse model for retinal acute injury, SI (Sigma, 7681552) (30 mg/kg body weight) was injected intraperitoneally 7 days later. Same age mice injected with PBS intraperitoneally as the control group.

### ONL thickness analysis

Photomicrographs were obtained by tiling up of six individual micrographs stained with DAPI at × 200 magnification. Three sections passing through the optic papilla were chosen from each eye, and at least three mice were included in each group. To measure the thickness of the ONL, the optic nerve head was defined as the original location (recorded as 0) and four positions were uniformly selected at both the temporal and the nasal side of the retina. The ONL thickness was calculated based on its vertical row numbers measured using ImageJ (1.42) software.

### Electroretinogram recording

Visual function was tested by the standard protocol of the ISCEV with a full-field flash ERG system (PuREC and LED Visual Stimulator LS-100/200, Mayo Corporation, Aichi, Japan), according to the manufacturer’s protocol. Mice were kept in a dark room overnight for dark adaptation. All operations were performed under dim red light. Mice were anesthetized by intraperitoneal injection of pentobarbital sodium (1%) (7 μl/g body weight). After anesthesia and mydriasis placed earth along the tail, put a reference electrode into the mouth, and placed a clip contact lens electrode on the corneal surface. Then the a-wave and the b-wave amplitude were recorded using an RETI-Port device and measured with different flash intensities. The a-wave amplitude was measured as the maximum negative trough below the baseline, the b-wave amplitude was calculated from the a-wave trough to the maximum subsequent positive peak, and the signal-to-noise ratio was improved by interstimulus intervals. The scotopic combined rod-cone response 3.0 cd s m^−2^ was shown in this study.

### Tissue preparation and immunohistochemistry for frozen section

Mice were killed using CO_2_ at a 30% chamber replacement rate. After the mice had been completely euthanized, their eyes were removed. The cornea was removed after the eyes were soaked in 4% paraformaldehyde (PFA) for 20 min. After 1.5 h in PFA, the eye cups were put in 30% sucrose overnight at 4 °C. After removing the lens, the eye cups were air dried and imbedded with ideal cutting temperature compound, 4 °C freezing for 30 min, and stored at 20 °C until sectioned at 12 μm thickness.

The retinal slices were dried at room temperature for about 30 min before being washed three times (10 min each) with 1 × PBS (Solarbio, P1010), permeabilized for 10 min at room temperature with 0.3% Triton X-100 in 1 × PBS, and incubated with blocking solution (5% bovine serum albumin in 0.3% Triton) for 1 h at 37 °C. Primary antibodies were treated with the retinal slices overnight at 4 °C. The slices were then rinsed three times (10 min each) with 1 × PBS at room temperature before being incubated with secondary antibodies for 2 h at 37 °C. Sections were stained with DAPI for 7 min and cleaned as previously described. Finally, for sealing, an anti-fluorescence quenching agent was utilized. The working dilution ratio and sources of primary and secondary antibodies are listed in Additional file 1: Table S1.

### Tissue preparation and immunohistochemistry for whole-mount retina

Mice were killed using CO_2_ at a 30% chamber replacement rate. After the mice had been completely euthanized, their eyes were removed. The cornea was removed after the eyes were soaked in 4% PFA for 20 min. Following 1 h in PFA, the lens was removed; the eye cups were cut into four petals, similar to a four-leaf clover, then turned over and the retina was gently peeled out. The whole-mount retinas were washed three times (10 min each time) with 1 × PBS (Solarbio, P1010), permeabilized for 2 h at room temperature with 3% Triton X-100 in 1 × PBS, and incubated overnight at 4 °C with blocking solution (5% bovine serum albumin in 0.3% Triton). The retinas were then treated with primary antibodies overnight at 4 °C. After that, the sample was incubated with secondary antibodies for 2 h at 37 °C after being rinsed three times (10 min/time) with 1 × PBS at room temperature. To stain the nuclei, retinas were treated with DAPI for 10 min before being rinsed as before. The anti-fluorescence quenching agent was then used as a sealant. Additional file 1: Table S1 lists the sources of primary and secondary antibodies as well as the working dilution ratio for each.

### EdU incorporation assay

A 50 mg/kg intraperitoneal dose of EdU powder dissolved in PBS was given twice (12 h and 2 h before killing) at 1 dpi, as well as three times (24 h, 12 h, and 4 h before killing) at 3, 5, 14, 21 and 28 dpi in SI-injured groups. Of note, in AAV-infected groups, EdU was also given every day from 1dpi until 14dpi. A fluorogenic click reaction using the BeyoClick™ EdU Cell Proliferation Kit with Alexa Fluor 647 was used to identify the EdU-labeled cells. The percentage of EdU-labeled tdTomaoto positive MG in each area of the retina was computed and the numbers of EdU-labeled cells were manually counted.

### Western blot analysis

The retinas were stripped from the eyes and lysed with RIPA lysis buffer containing 1% PMSF. Equal amounts of total protein were separated by SDS-PAGE on precast gels (Nanjing ACE Biotechnology) for 25 min at 160 V and rapidly transferred onto polyvinylidene difluoride (PVDF) membranes for 20 min at 300 mA. The membranes were blocked using a quick blocking buffer for 10 min at room temperature and then incubated with primary antibodies overnight at 4 °C. Next, membranes were washed thrice for 10 min with TBST (TBS with 0.1% Tween 20) and incubated with secondary antibodies for 1 h at room temperature. Finally, protein bands were detected using an ECL luminescence kit (Mengbio) and imaged with a ChemiDoc Imaging system (Bio-Rad). The intensity of the bands was measured with ImageJ (1.42) software. The working dilution ratio and sources of primary and secondary antibodies are listed in Additional file 1: Table S2.

### Bulk-RNA library preparation and sequencing

Five groups were formed (control, 1, 3, 5, and 14 dpi). There were three replicas in each group. RNAiso Plus reagent (Takara, 9108, Japan) was used to extract total RNA in accordance with the manufacturer's instructions. For library creation and sequencing, retinal RNA samples were delivered to Gene Denovo Biotechnology Company in Guangzhou, China. An Agilent 2100 Bioanalyzer was used to evaluate RNA mass detection (Agilent RNA 6000 Nano Kit). Using the Illumina Novaseq 6000 system, total RNA was sequenced from the RNA samples with stranded and 150-bp paired-end reads; the length of the sequencing reads was 20 M; and the volume of the sequencing data was 6G.

### Definition of DEGs from bulk-RNA seq

Gene expression levels are shown as FPKM values. DEGs were detected by DESeq2, and identified based on the following criteria: false discovery rate (FDR) < 0.05, |fold change| ≧ 2 (Fig. 2); false discovery rate (FDR) < 0.05, |fold change| ≧ 1.5 (Fig. 5).

### GO terms and KEGG enrichment analysis

All DEGs were mapped to GO terms/KEGG in the GO/KEGG database for biological functions analysis. The significantly enriched GO terms/KEGG in DEGs compared with the genome background were defined.

### Protein–protein interaction

Protein–protein interaction network of the obtained significantly enriched DEGs was constructed using String v10, where genes are visualized as nodes and interactions are visualized as lines. The network file was visualized using Cytoscape (v3.7.1) software to present biological core and hub gene interactions.

### Tissue dissociation and single-cell suspension preparation for single-cell RNA seq

Three groups were formed (control, SI-3d and SI-7d). SI-3d and SI-7d groups each had three replicates, while the control group only had two. All operations were performed on ice. The retinas were taken from the eyeballs, immersed in PBS, and placed in pre-chilled DMEM + 1% FBS to dissociate. Then the retinas were cleaned with PBS containing 0.05% BSA. The retinas were digested with 2 mL GEXSCOPE™ Tissue Dissociation Solution (Singleron, China) and dissociated by shaking at 180 rpm for 8 min at 37 °C in a constant-temperature shaker. After digestion, the retinas were filtered with a 40-μm filter without lysis of red blood cells. The lysate was centrifuged at 300 rpm for 5 min at 4 °C to collect the cells. The cell pellet was resuspended in 500 μl 10% FBS in DMEM and cells were counted.

### scRNA seq library, quantification, and statistical analysis

Retinal cells were suspended with PBS (Boster, PYG0021) at a concentration of 1 × 10^5^ cells/ml. Then, the suspension was loaded onto a microfluidic device, and GEXSCOPER Single-Cell RNA Library Kit was used to establish the scRNA seq libraries according to the manufacturer’s protocol (Singleron Biotechnologies). Individual libraries were diluted to 4 nM and pooled for sequencing on an Illumina NovaSeq 6000 with 150-bp paired-end reads. Raw reads were processed with an internal pipeline to generate gene expression profiles. After filtering read 1 without poly T tails, the cell barcode and UMI were extracted. Adapters and poly-A tails were trimmed (cutadapt 1.17) before aligning read 2 to mus_musculus_ensembl_92 with ensemble version 92 gene annotation (cutadapt 1.17 and featureCounts 2.0.1) [26]. Reads were grouped according to a median unique molecular identifier (UMI) count of 899–3402 per cell, or a mean depth of 442,348,052 reads per library. RNA seq data were analyzed with the Seurat (v.3.4.2) R package (http://satijalab.org/seurat/), including cell type analysis and clustering analysis [27]. The read table function was used to load UMI count tables into R. For clustering analysis, the parameter resolution was set at 0.8 for the FindClusters function.

### Identification of retinal cell types

Cell types were classified using differential expression analysis, which compared each cluster with all others combined using the Wilcoxon method in Seurat to identify cluster-specific marker genes. Each retained marker gene was expressed in a minimum of 25% of cells and had log_2_ (fold change) ≥ 0.25. In our paired cluster analysis, DEGs were considered significant if *P*_adj_ < 0.01 (Benjamini–Hochberg correction for multiple testing) and | log_2_ (fold change) |≥ 0.5.

### Dissecting temporal sequential mRNA expression of MG

The cells annotated as MG in the first round (main ACTIONet) was extracted and a new count matrix was created. This count matrix was then batch-corrected, reduced with the ACTION kernel, and reassembled as a subACTIONet for MG. The final MG subACTIONet had 2537 filtered/batch-corrected MG in nine distinct clusters.

### Trajectory analysis

R package Monocle (version 2.4.0) was used to reconstruct differentiation hierarchies within diverse clusters of MG [28]. Monocle 2 package was used to construct cell fate decisions and differentiation trajectories, which generated a pseudotime plot that could account for both branched and linear differentiation processes by applying reverse diagram embedding based on a user-defined gene list.

### RNA extraction and RT-qPCR

Total RNA was extracted from intact retina using a traditional method. Retinas were placed in 200 μl of chloroform and 1 ml RNAiso Plus (Takara, 9108, Japan), shaken vigorously, and centrifuged at 12,000 rpm for 5 min (4 °C). The supernatant was transferred to a sterile EP tube, mixed with 500 μl isopropanol, and stored at -20 °C overnight. The solution was centrifuged at 12,000 rpm for 15 min at 4 °C and the supernatant was discarded. Next, 1 ml of 75% alcohol was added to each tube, samples were centrifuged at 7500 rpm for 5 min at 4 °C, and the supernatant was removed. After another centrifugation step at 12,000 rpm for 15 s, the supernatant was removed. Samples were dried at room temperature until alcohol had fully evaporated. Then 20 μl DEPC water was added to dissolve RNA. The RT-qPCR assays were performed using a Prime Script RT-qPCR Kit (Takara) on a Real-Time instrument (Bio-Rad). The Primers used in this study are listed in Additional file 1: Table S3.

### AAV production and intravitreal injection

Mouse Hmga2 cDNA, reverse-transcribed and amplified from retinal RNA, was subcloned and inserted into an AAV vector backbone where the expression was driven by the *GFAP* promoter (a gift from Mengqing Xiang, Sun Yat-sen University). Individual AAV was produced in AAV-293 cells (Procell Co., Ltd., Wuhan, China) by plasmid co-transfection and iodixanol gradient ultracentrifugation. Purified AAVs were concentrated with Amicon Ultra-15 Filter Units from Millipore and the viruses were further concentrated by reducing the volume to a final titer of 1.0–5.0 × 10^11^ genome copies per ml. Virus titers were determined by RT-qPCR using linearized plasmid standards and primers against the inverted terminal repeat (ITR). The AAVs without Hmga2 were used as the control AAVs. 2 µl per eye for AAVs were injected intravitreally. After injecting AAV into the mouse retina, the mouse eye surface was coated with recombinant bovine basic fibroblast growth factor and tobramycin dexamethasone eye ointment, and the mouse was put on a heating plate for rewarming.

### Light/dark transition test

The testing for the light/dark transition was carried out as previously mentioned [29]. Two-thirds of the box's volume was a light zone, with a conduit allowing mice to move freely between the two. One-third of the box’s volume was a dark zone that was enclosed before the experiment started. In the space directly above the light zone, a light-emitting diode with a light intensity of 300–400 lx was hung. The mice were initially kept in a dark area for 2 min. Next, after opening the channel, a camera was used to film the mice's activity in the box for 10 min.

### Statistical analysis

All data were shown as the mean ± SD. Statistical analysis of data was performed by nonparametric one-way ANOVA or Unpaired *t*-test using SPSS V25.0 software. *P* < 0.05 was considered significant. Graphs were drawn using Prism 6.01.

## Results

### MG gliosis fate determination occurred at 3–5 days after sodium iodate-induced retinal acute injury

A model for acute retinal injury was created by sodium iodate (SI) injection utilizing MG lineage-tracing mice. The harvest time points in this assay were set at 1, 3, 5, and 14 days post injection (dpi) (Additional file 1: Fig. S1a). SI injection led to local RPE loss followed by photoreceptor degradation was also widely utilized to establish AMD and RP model [30, 31]. The fine morphological details of MG were identified in MG lineage-tracing mice (Additional file 1: Fig. S1b), and tdTomato positive cells were completely co-labeled with MG, but failed to co-label with photoreceptors and ganglion cells (Additional file 1: Fig. S1c, d). After 1 day of SI injection, there were obvious alterations in the number of outer nuclear layer (ONL) cell rows and a- and b-wave amplitudes, but the arrangement of the photoreceptor segments was chaotic. At 3 and 5 dpi, both the number of ONL cell rows and a- and b-wave amplitudes significantly decreased (*P* < 0.001). At 14 dpi, the number of ONL cell rows decreased to 6–7 and scotopic responses were almost nonexistent (*P* < 0.001) (Additional file 1: Fig. S1e–h).

**Fig. 1:**
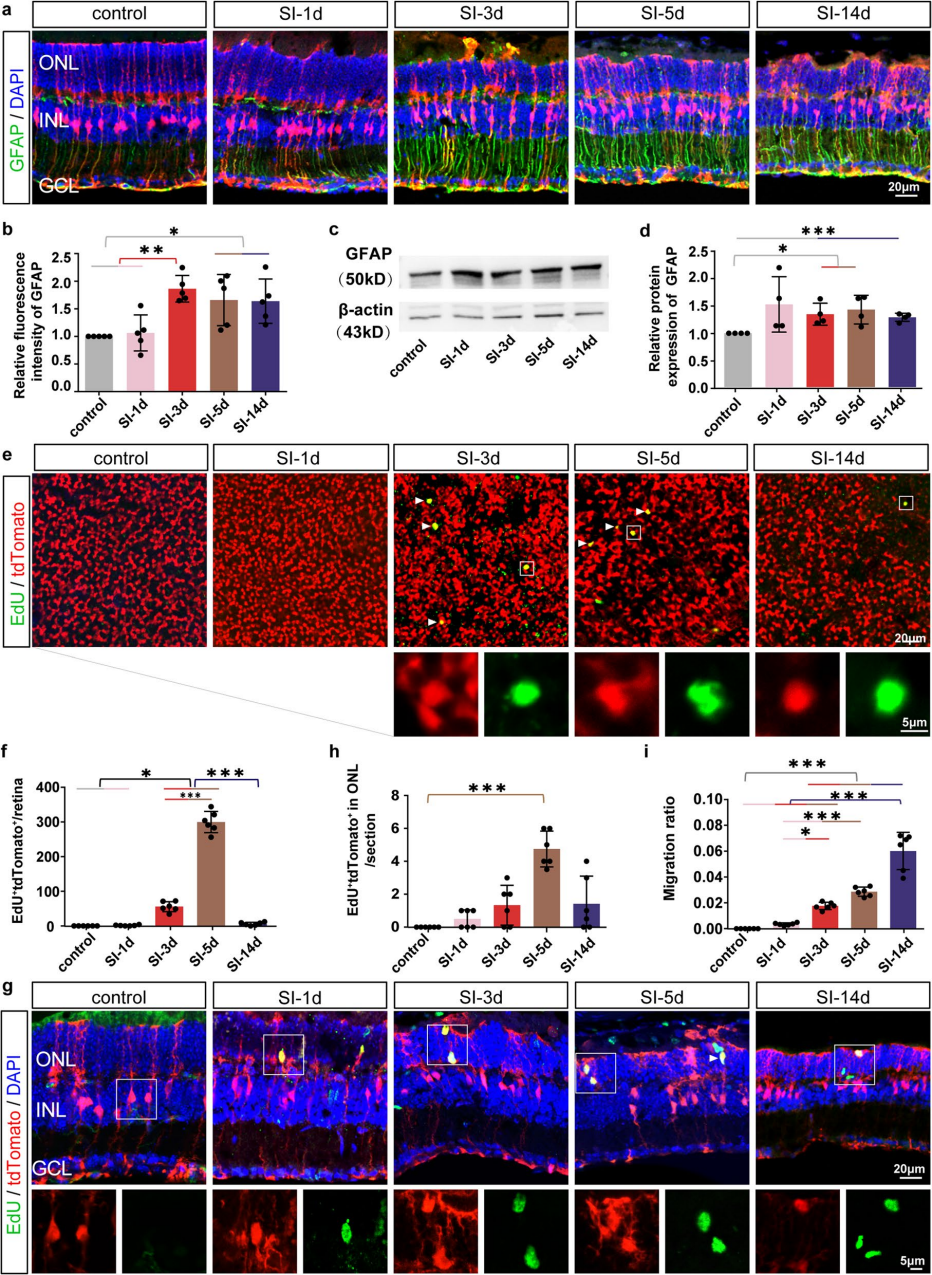
MG gliosis fate determination occurred at 3–5 days after sodium iodate-induced retinal acute injury. **a** Immunohistochemical labeling for GFAP, tdTomato and DAPI in the central retinas of control and SI-treated groups. **b** Relative fluorescent intensity GFAP protein in control and SI-treated retinas over time (*n* = 5). **c**, **d** Western blot analysis of GFAP protein expression in control and SI-treated retinas (*n* = 4), full-length gels and blots are presented in Additional file 1: Fig. S8. **e** Representative whole-mount retina partial images of tdTomato and EdU staining. Insets display the enlarged drawings of each fluorescent reporter (White Square). White solid arrows identify the tdTomato and EdU double positive cells. **f** Ratio of EdU and tdTomato double positive cells in each whole-mount retina of control and SI-treated groups (*n* = 6). **g** Representative retinal section partial images of tdTomato, EdU and DAPI staining. Insets display the enlarged drawings of each fluorescent reporter in EdU and tdTomato (White Square). White arrows identify the EdU and tdTomato double positive cells. **h** Ratio of EdU and tdTomato double positive cells in the ONL of control and SI-treated retinas (*n* = 6). **i** Ratio of migrated MG in control and SI-treated retinas (*n* = 6). *ONL* Outer nuclear layer, *INL* Inner nuclear layer, *GCL* Ganglion cell layer. *P < 0.05; **P < 0.01; ***P < 0.001, one-way ANOVA test was used in (**b**, **d**, **f**, **h**, **i**)

To confirm the time window for the MG gliosis fate determination, we firstly observed GFAP expression after acute injury. In the normal retina, GFAP was mostly expressed in the inner half of the retinal MG and their end feet terminals close to the ganglion cell layer (GCL). GFAP basal processes were overexpressed in MG and extended into the inner plexiform layer at 1 dpi. From 3 to 14 dpi, the GFAP positive fibers then penetrated well into the apical processes of the ONL, the expression of GFAP protein was increased compared with control group (Fig. 1a, b). The greatly increased expression at the appropriate time points after retinal injury was validated by Western blot analysis of GFAP protein levels (*P* < 0.01) (Fig. 1c, d). These results revealed that MG gliosis gradually aggravated from 1 to 3 dpi, and reached its peak at 3 dpi.

MG proliferation may contribute to establishing glial scar, or be an indicator of cellular de-differentiation for MG reprogramming [10, 16]. We utilized EdU labeling to monitor proliferative MG in vivo during retinal injury process. It demonstrated that only a small portion of MG were EdU-labeled in the retina at various time points. The number of EdU positive MG increased progressively starting at 3 dpi, peaked at 5 dpi, and then reduced at 14 dpi (Fig. 1e, f). The low and transient proliferation of activated MG may imply the low potential of de-differentiation for MG reprogramming.

MG migration always takes place at the late stage of gliosis development [10, 32]. From 3 to 14 dpi, we observed that some MG moved into the ONL which were also EdU positive, and the amount of migrated MG was gradually increased (Fig. 1g–i), suggesting a low and transient migratory capacity in the activated MG by SI.

Taken together, we supposed that the 3-5dpi was a crucial time window for MG gliosis fate determination in models with SI injury.

### The most obvious transcriptional alterations of retina and Müller glia occurred at 3 days post-injection

To further confirm the time point for MG gliosis fate determination during retinal injury and explore potential reprogramming molecular targets, bulk-RNA seq and scRNA seq were utilized to examine the transcriptional alterations of the retina and MG following SI injury.

**Fig. 2:**
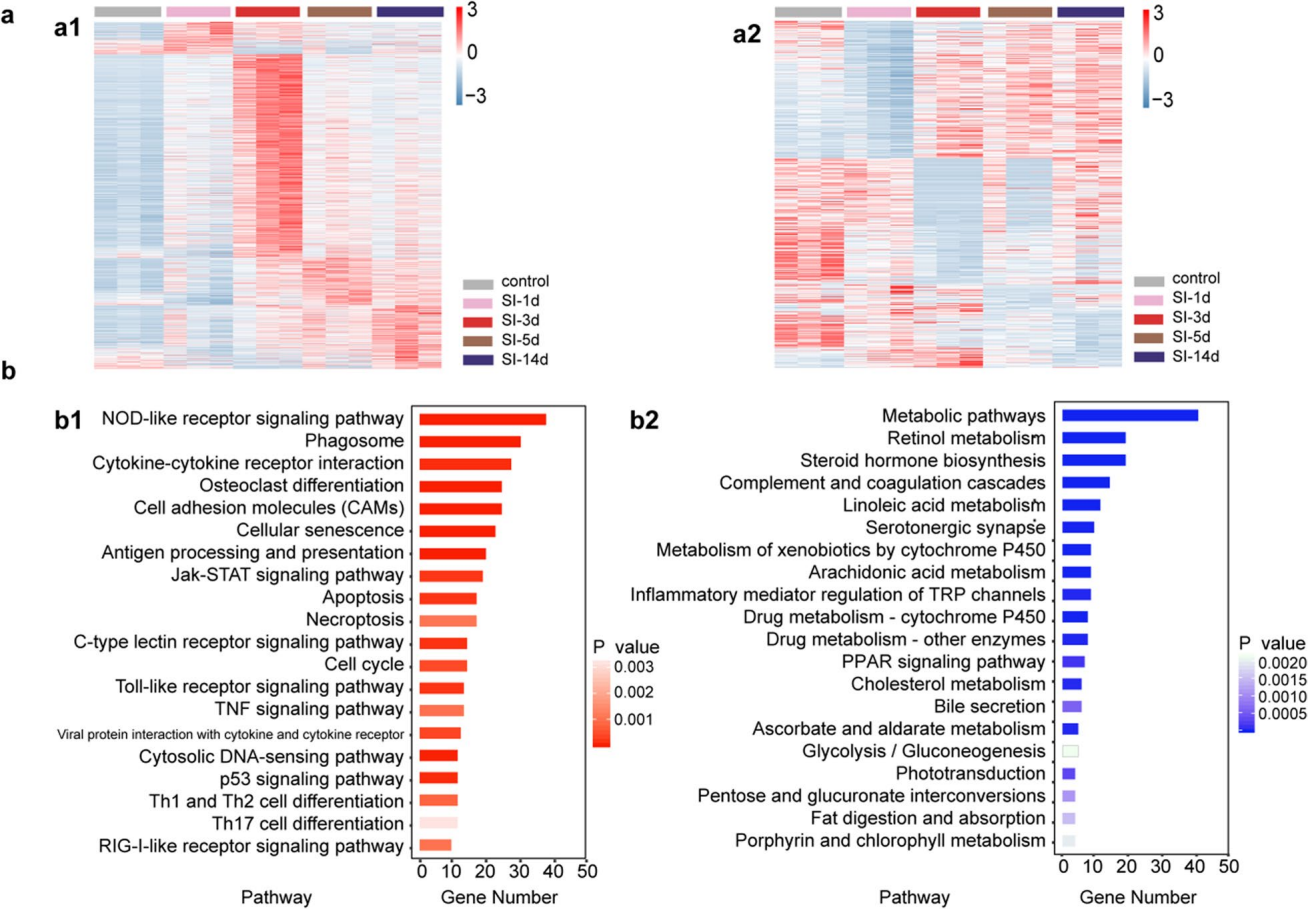
Response of retina to acute retinal injury at 3 days post-injection. **a** In the heatmap, the relative expression level (FPKM values were normalized) of up-regulated (a1) or down-regulated (a2) DEGs that changed the greatest at a specific injury group (SI-1d, SI-3d, SI-5d or SI-14d) as compared with other groups. The colors represent the relative expression quantities. **b** The top 20 pathways of up-regulated (b1) or down-regulated (b2) DEGs in the retina that changed the greatest at 3dpi compared with other groups. The colors red and blue signify up-regulated and down-regulated pathways, respectively

The bulk-RNA seq library was firstly created from the retina at various time points following SI injury. 2128 DEGs were screened out in the injury groups against control group. Then, we compared the gene expression at one particular injury time point with all the other injury time points. The highest up- and down-regulated DEG counts were observed at SI-3d (Fig. 2a).

At 3 dpi, the toxic harm brought on by SI injection was confirmed by the activation of the NOD-like receptor signaling pathway, phagosome, cellular senescence, and apoptosis (Fig. 2b1). The cell cycle and the JAK/STAT signaling system, which are linked to MG reprogramming were both engaged (Fig. 2b1), highlighting the mouse retina’s potential and momentary reprogramming capabilities [33]. Moreover, down-regulated DEGs were primarily connected to phototransduction, serotonergic synapses, and retinol metabolism, indicating alterations in retinal function (Fig. 2b2).

Then, we isolated single retinal cells at 3 dpi and 7 dpi, which separately corresponded to the crucial time point and the late stage of acute injury, and performed a scRNA seq analysis on the acutely injured mouse retinas. In total, 54,236 individual retinal cells from three groups (control, SI-3d, and SI-7d) were profiled. Canonical markers for the cell types of the retina were used to identify ten cell clusters. Rod cells, cone cells, MG, bipolar cells, amacrine cells, microglia, RPEs, and horizontal cells were identified as the primary retinal cell types (Additional file 1: Fig. S2a, b). 4,007 MG cells were identified among them.

By contrasting a particular time point (3 dpi or 7 dpi) with the control group, the number of up- or down-regulated DEGs was determined using single-cell expression profiles of MG (Additional file 1: Fig. S2c). GO analysis revealed that the most prevalent terms at 3 dpi were associated with the cilium of the photoreceptor cell, viral response, and nucleoside binding (Additional file 1: Fig. S2d, e).

### Temporal sequential response of Müller glia to acute retinal injury

**Fig. 3:**
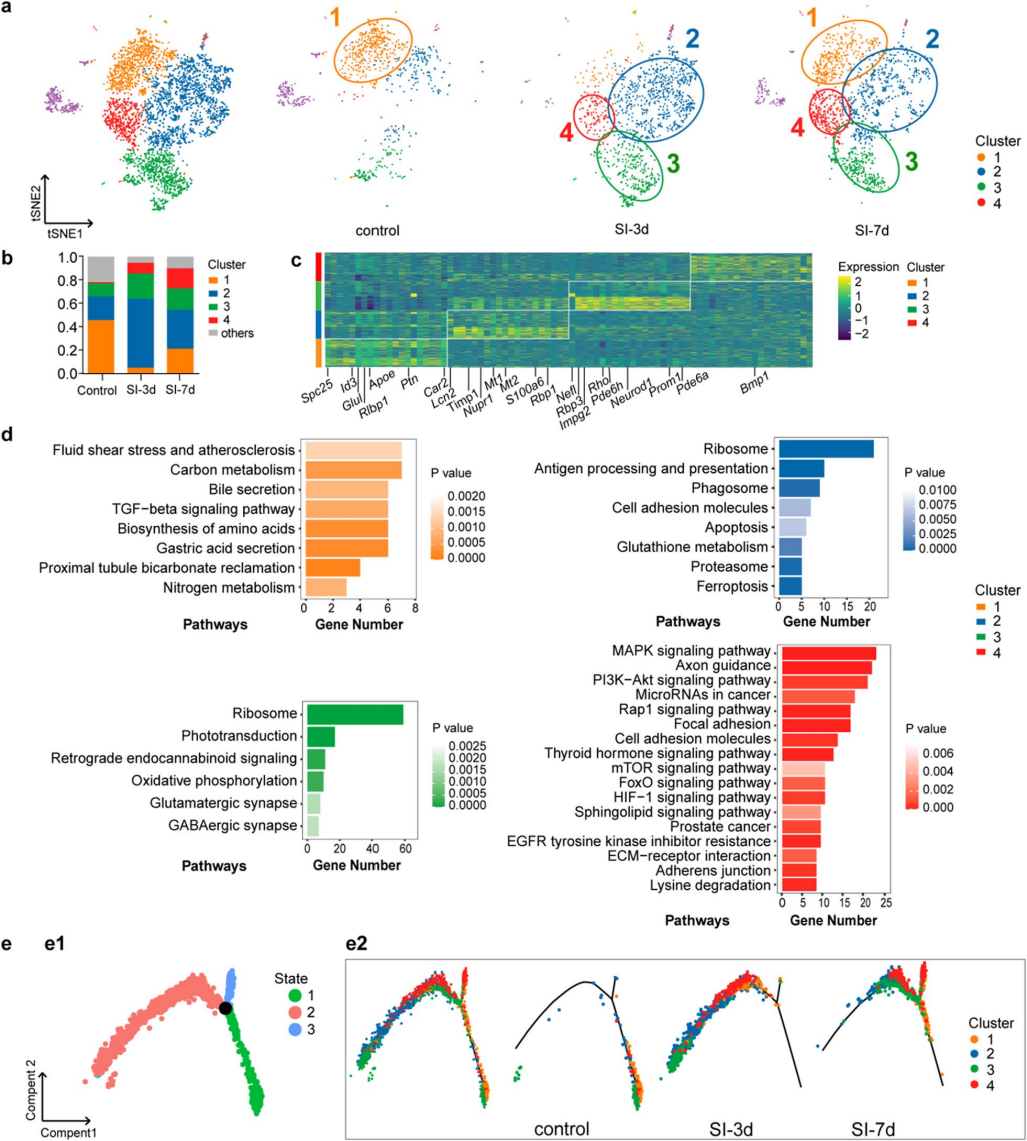
Temporal sequential response of Müller glia to acute retinal injury. **a** t-SNE plots show primary four clusters of MG in control and SI-treated groups, clusters 1–4 were framed with circles of different colors. **b** Percentages of clusters 1–4 and others of MG in control, SI-3d and SI-7d groups. **c** Top20 DEGs’ expression in clusters 1–4 of MG. **d** Enriched KEGG pathways based on DEGs in clusters 1–4 of MG. **e** Trajectory analysis of three states (e1) and dynamic distribution in clusters 1–4 of control and SI-treated MG (e2)

We re-clustered all MG into nine clusters in order to highlight the temporal sequential transcriptional alterations of MG after injury; the top four clusters accounted for the majority of the cells. Clusters 2, 3, and 4 were primarily formed in the injury groups (SI-3d and SI-7d), whereas cluster 1 was primarily enriched in the control and SI-7d group, which represented a relative resting state (Fig. 3a). In the control group, 45.9% of the MG belonged to cluster 1. At 3 dpi, MG evolved into three subpopulations: cluster 2 (58.8%), cluster 3 (21.4%), and cluster 4 (9.1%). The ratio of MG homeostasis genes in cluster 1 was drastically reduced, down to 5.4% at 3pi and rebound to 21.5% at 7dpi (Fig. 3b).

In cluster 1, representative DEGs included *Glul*, *Rlbp1* and *Apoe* (Fig. 3c), and several metabolic processes involved in maintaining homeostasis, such as carbon metabolism, nitrogen metabolism, and TGF-β signaling, were filtered out by KEGG analysis (Fig. 3d). In cluster 2, *Lcn2*, *Timp1* and *Nupr1* were concentrated (Fig. 3c), and injury-related pathways (apoptosis, phagosomes and ferroptosis) were shown to be significantly expressed (Fig. 3d). In cluster 3, DEGs involved in the operation of the retinal system, including the neuronal regeneration gene *Neurod1* and the photoconduction-related genes *Rbp3*, *Rho*, *Rde6h* and *Rde6a* were concentrated (Fig. 3c), and the phototransduction and synapse pathways were active. It was important to note that MG-derived progenitor cells’ endocannabinoid signaling, which enhances de-differentiation and proliferation was also active (Fig. 3d) [34]. The MAPK signaling route, thyroid hormone signaling pathway, PI3K/AKT signaling pathway, and mTOR signaling pathway were among the signal transduction pathways abundant in cluster 4 and were known to control MG reprogramming (Fig. 3d) [35–39].

Moreover, we created the injurious and pseudotime trajectories of MG states. State 1 of the MG comprised primarily clusters 1 and 3 in the control group, state 2 primarily contained clusters 2, 3, and 4 in the SI-3d group, and state 3 primarily contained cluster 4 in the SI-7d group (Fig. 3e). According to the trajectory study, MG started a string of reactions after retinal injury. Genes controlling MG cell activity (cluster 2) and retinal neuronal function (cluster 3) were active in the early stages of the injury (3 dpi). Part of the MG eventually returned to the homeostatic stage (cluster 1) as the injury increased, while other parts were continuously engaged in signal transduction (cluster 4).

Thus, we identified four major MG clusters and three states that dynamically responded to retinal injury. The majority of MG were activated after retinal injury (cluster 2), a portion of them showed neurogenic potential and related gene expression profiles (clusters 3, 4), and some of them returned to resting at 7 dpi. The four major MG clusters screened out in the present study implied that not only injury response, but also transient cell reprogramming was initiated after acute injury to the retina. Extending the duration of the cell reprogramming or stimulating the reprogramming of specific MG subpopulations might be an effective regenerative strategy in the injured retina. It was demonstrated that two clusters (clusters 1 and 3) in the control group exhibited different trajectories, and the subpopulation of MG cells in cluster 3 was more sensitive than those in cluster 1. Whether the MG subpopulation in these clusters responds differently or maintains a prominent reprogramming capacity in the retina of mice remains to be explored.

### Screening and identifying Hmga2 as an epigenetic regulator for Müller reprogramming process

**Fig. 4:**
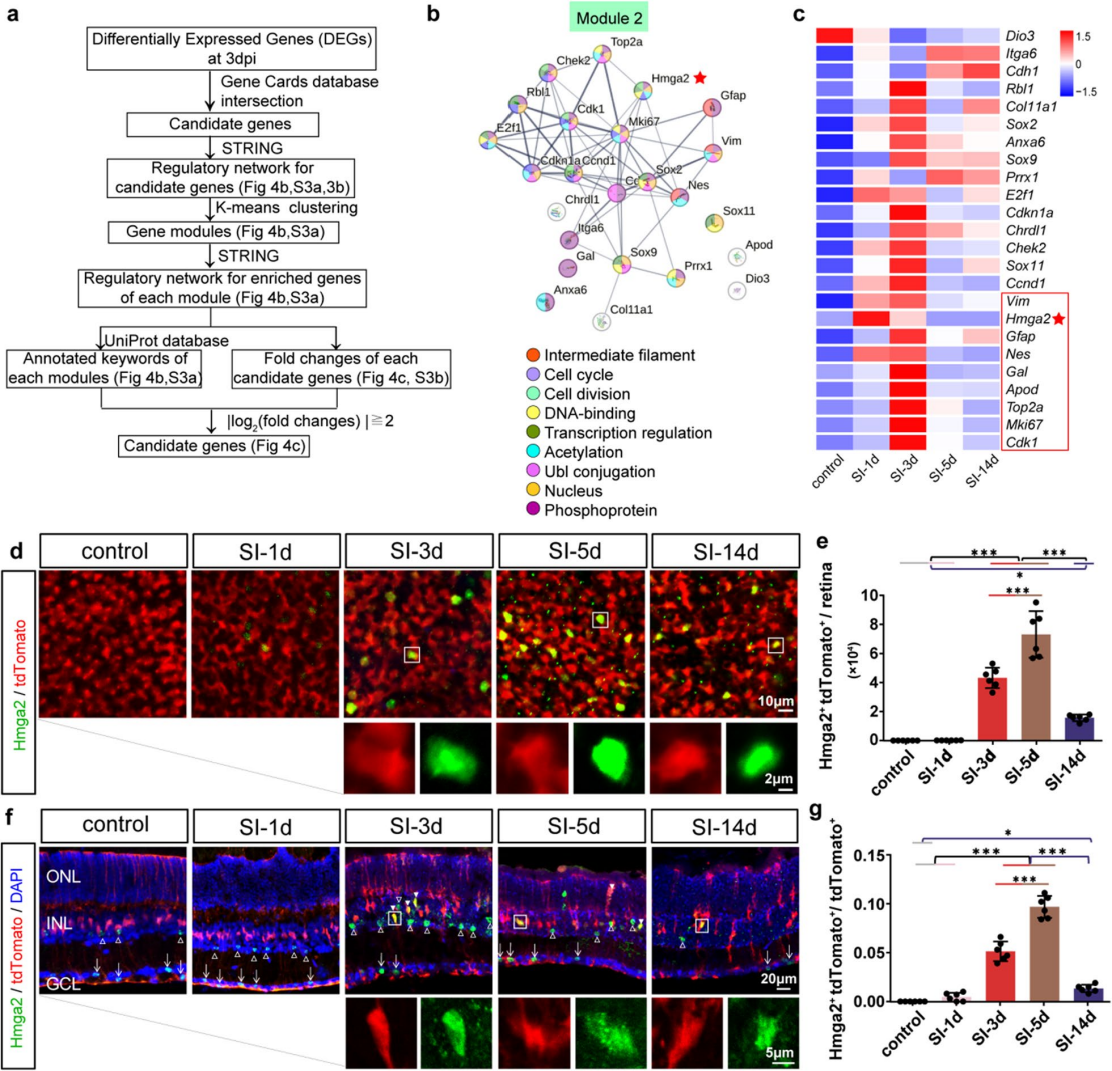
Screening and identifying Hmga2 as an epigenetic regulator for Müller reprogramming process. **a** Workflow for screening the potential genes for MG reprogramming process among DEGs from bulk-RNA seq and scRNA seq. STRING Software, Gene Cards database and UniProt were used in screening. **b** UniProt database annotations for keywords of module 2. Line thickness represents the degree of data support; a minimum interaction score of 0.5 is needed. **c** Relative expression of screened 24 DEGs of module 2 in each group. The colors correspond to relative expression. Red framed 9 candidate regulators with log_2_ (fold change)  ≧ 2 at 3dpi. **d** Representative whole-mount retina partial images with tdTomato and Hmga2 staining. Insets display the enlarged drawings of each fluorescent reporter (White Square). **e** Ratio of Hmga2 and tdTomato double-positive cells in control and SI-treated whole-mount retinas (*n* = 6). **f** Representative retinal section partial images of tdTomato, Hmga2 and DAPI staining. Insets display the enlarged drawings of each fluorescent reporter in tdTomato and Hmga2 (White Square). White solid arrows identify the double positive cells. White hollow arrows identify the Hmga2 positive and tdTomato negative cells in INL. White arrowheads indicate Hmga2 positive cells in GCL. **g** Ratio of Hmga2 and tdTomato double positive cells in each retinal section of control and SI-treated groups (*n* = 6). *ONL* Outer nuclear layer, *INL* Inner nuclear layer, *GCL* Ganglion cell layer. *P < 0.05, **P < 0.01, ***P < 0.001, one-way ANOVA test was used in (**e**, **g**)

To evaluate potential epigenetic regulators in MG reprogramming, we established a screening workflow (Fig. 4a). We chose the terms relating to the MG reprogramming process, including “dedifferentiation”, “cell cycle”, “proliferation”, “neurogenesis”, “epigenetic” and “Müller”, and downloaded their gene sets from the Gene Cards database. These gene sets had a gene number of 1616, 17,153, 18,411, 3240, 16,541 and 7006 respectively. Subsequently, two subsets with 79 DEGs and 10 DEGs were created by intersecting these gene sets with DEGs from bulk-RNA seq (SI-3d) and scRNA seq (SI-3d), respectively.

Next, we created a protein–protein interaction network of the 79 DEGs, and divided them into two modules through the k-means clustering approach. The number of DEGs in module 1 and module 2 was 55 and 24, respectively. Using the UniProt database, keywords were annotated for each module. Based on the correlation strength, the keywords for module 1 were mainly “Mitogen”, “Proteoglycan”, “Alternative initiation”, “Growth factor”, “Biological rhythms” and “Angiogenesis” terms. *Fgf2* was the hub gene with three biological roles in comparison to other genes (Additional file 1: Fig. S3a). Similarly, the keywords in module 2 were intermediate filament, cell cycle, cell division, DNA-binding, etc. *Hmga2* was the hub gene with seven biological functions in comparison to other genes (Fig. 4b). The 10 DEGs we got from scRNA seq were also formed a protein-protein interaction network, but their direct contact was very poor (Additional file 1: Fig. S3b). Moreover, the |log_2_ (fold changes)| values of these 10 DEGs were all below 2 at 3 dpi (Additional file 1: Fig. S3c).

We considered the protein–protein interaction network and the value of log_2_ (fold changes) to determine the final candidate regulator for the MG reprogramming process. Nine DEGs (*Vim, Hmga2, Gfap, Nes, Gal, Apod, Top2a, Mki67* and *Cdk1*) with |log_2_(fold changes)|  ≧ 2 at 3 dpi in module 2 were selected out (Fig. 4c). Among these 9 DEGs, Hmga2 owned the most reprogramming-related functions (Fig. 4b).

In addition, Hmga2, the most prevalent nonhistone chromatin-associated protein, is abundantly expressed in embryonic stem cells during embryogenesis while lowly expressed later in development and in adulthood [40]. Hmga2 is essential for controlling gene transcription to control stem cell destiny, neurogenesis [41–50]. The analogous gene of *Hmga2*, *hmga1a*, was up-regulated in reactive MG and enhanced proliferation and neurogenesis in zebrafish, as demonstrated in the previous work [48]. Thus, we decided to examine *Hmga2* in more detail.

Using RT-qPCR, we verified that the mRNA expression trends of *Hmga2* and other partial genes, such as *Igfbp3*, *Stat3*, *Tgfb1* and *Ccnd1,* were consistent with that in bulk-RNA seq (Additional file 1: Fig. S4a). Transcriptional expression of *Hmga2* in MG was also validated by scRNA seq, it revealed that Hmga2 was almost not expressed in normal retina, and lowly expressed in injured MG (SI-3d, SI-7d). Only 0.90% MG were tested to transcribe Hmga2 at 3dpi (Additional file 1: Fig. S4b).

The transcriptional expression of *Hmga2* in the whole retina considerably increased at 1dpi and 3dpi when compared to that of the intact control, with the log_2_ (fold changes) ≧ 2 (Fig. 4c). However, there was no significant difference of the number of Hmga2 protein positive MG between the SI-1d and control groups. From 3 to 5 dpi, the number of Hmga2 protein positive MG increased gradually, from 4 × 10^4^ to 8 × 10^4^ per retina, the proportions of tdTomato positive MG were 5% and 10%, respectively (Fig. 4d–g). At 5 dpi and 14 dpi, the transcriptional level of *Hmga2* in the whole retina showed no marked changes, with the log_2_ (fold changes)≦0.02 (Fig. 4c). At 14 dpi, the number of Hmga2 protein positive MG dropped significantly, to just 1.6 × 10^4^ per retina, the proportion of tdTomato positive MG was around 2% (Fig. 4d–g). It indicated that there was only a temporary up-regulation of the Hmga2 protein in MG after the retina was injured. Moreover, some Hmga2 positive cells were found in the GCL at each group. Few Hmga2 positive cells were also found in the ONL at 5 dpi (Fig. 4f).

The expression of *Hmga2* through bioinformatics was verified by RT-qPCR, and the expression of Hmga2 protein was verified by immunohistochemical staining. The transcriptional level and protein level of Hmga2 showed coordinated changing tendency during the different time points of retinal injury, while protein expression of Hmga2 lagged after transcriptional changes, possibly because it takes time for transcription to translate into protein.

### Hmga2 overexpressing in MG down-regulated retinal gliosis related genes and up-regulated photoreceptor survival related genes

To explore functions of Hmga2 in retina after SI injury, mice aged 5–6 weeks received intravitreal injections of either control adeno-associated virus (AAV) or AAV carrying Hmga2 (experimental AAV), and SI was injected 20 days after that. The harvest time points were 14 dpi, 21 dpi and 28 dpi (Additional file 1: Fig. S5a). A typical arbor-like morphology was displayed by the AAV-infected cells, precisely as it had been presented with the MG lineage-tracing mice previously (Additional file 1: Figs. S5b, S1b). Besides, nearly all of the virus-infected MG were co-labeled with Sox9 (Additional file 1: Fig. S5c, d), and MG exposed to the experimental AAV expressed the Hmga2 protein, while MG exposed to the control AAV did not (Additional file 1: Fig. S5e, f), it indicated that experimental AAV targeted MG appropriately and overexpressed Hmga2 protein.

**Fig. 5:**
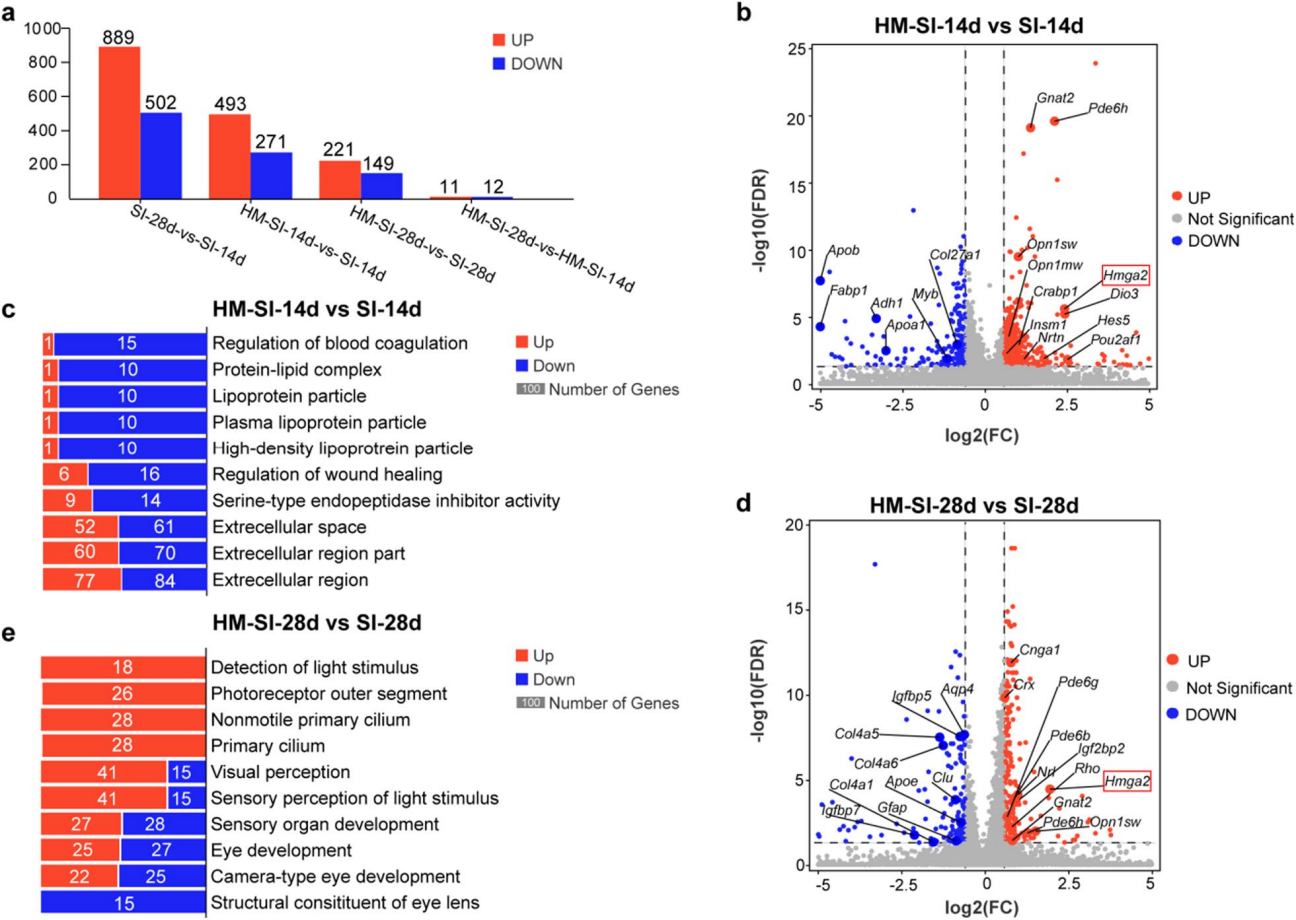
Hmga2 overexpressing in MG down-regulated retinal gliosis related genes and up-regulated photoreceptor survival related genes. **a** The number of up- or down-regulated DEGs between different two groups. **b** Representative DEGs from the HM-SI-14d group compared with the SI-14d group. **c** Top 10 GO terms in HM-SI-14d group compared with the SI-14d group. **d** Representative DEGs from the HM-SI-28d group compared with the SI-28d group. **e** Top 10 GO terms in HM-SI-28d group compared with the SI-28d group

To further explore functions of Hmga2 for retina after SI injury, bulk-RNA seq was performed on three additional groups (SI-28d, HM-SI-14d and HM-SI-28d), and the results were integrated with the sequencing data of the prior two groups (control and SI-14d) to produce new DEG sets. The number of DEGs in HM-SI-14d and HM-SI-28d compared to SI-14d and SI-28d were 764 and 370 respectively, while the number of DEGs in HM-SI-28d compared to HM-SI-14d was 23 (Fig. 5a).

The average log_2_(fold change) of Hmga2 in HM-SI-14d and HM-SI-28d groups was 2.43 and 1.95 respectively, which was closed to the fold change in SI-3d (Fig. 5b, d) (Additional file 1: Table S4). In HM-SI-14d group, the photoreceptor-related genes were up-regulated, such as *Pde6h*, *Gnat2*, *Opn1sw* and *Opn1mw*; the lipid metabolism-related genes were down regulated, such as *Apoa1*, *Apob* and *Fabp1* when compared with SI-14d group (Fig. 5b). GO analysis revealed that the top 10 terms in HM-SI-14d group were related to extracellular region and lipoprotein particle, almost of them were relatively down-regulated (Fig. 5c). In HM-SI-28d group, more photoreceptor-related genes were up-regulated, such as *Pde6h, Pde6b, Rho, Nrl, Crx Gnat1, Gnat2, Opn1sw* and *Opn1mw*; while glia-related genes, such as *Gfap, Clu, Aqp4* and *Apoe*, were down-regulated. Moreover, extracellular matrix-related genes, such as *Col4a6, Col4a5* and *Col4a1*, were also down-regulated when compared with SI-28d group (Fig. 5d). In HM-SI-28d group, the top 10 terms were mainly related to visual perception, eye development, detection of light stimulus, photoreceptor outer segment and cilium, and most of them were up-regulated (Fig. 5e). Of note, other gliosis-related gene, *Vimentin,* was also down-regulated by 0.78 and 0.70 times in HM-SI-14d and HM-SI-28d groups respectively. Cone marker, *arrestin*, was also up-regulated by 1.40 times in HM-SI-28d group when compared with SI-28d group, while it had no significant difference in HM-SI-14d group when compared with SI-14d group (Additional file 1: Table. S4).

The transcriptome results revealed that Hmga2 overexpressing in MG could regulate the retinal immune activities, down-regulate the retinal gliosis and promote photoreceptor survival.

### Hmga2 overexpressing in MG reduced MG gliosis and promoted MG to obtain cone’s marker without proliferation

**Fig. 6:**
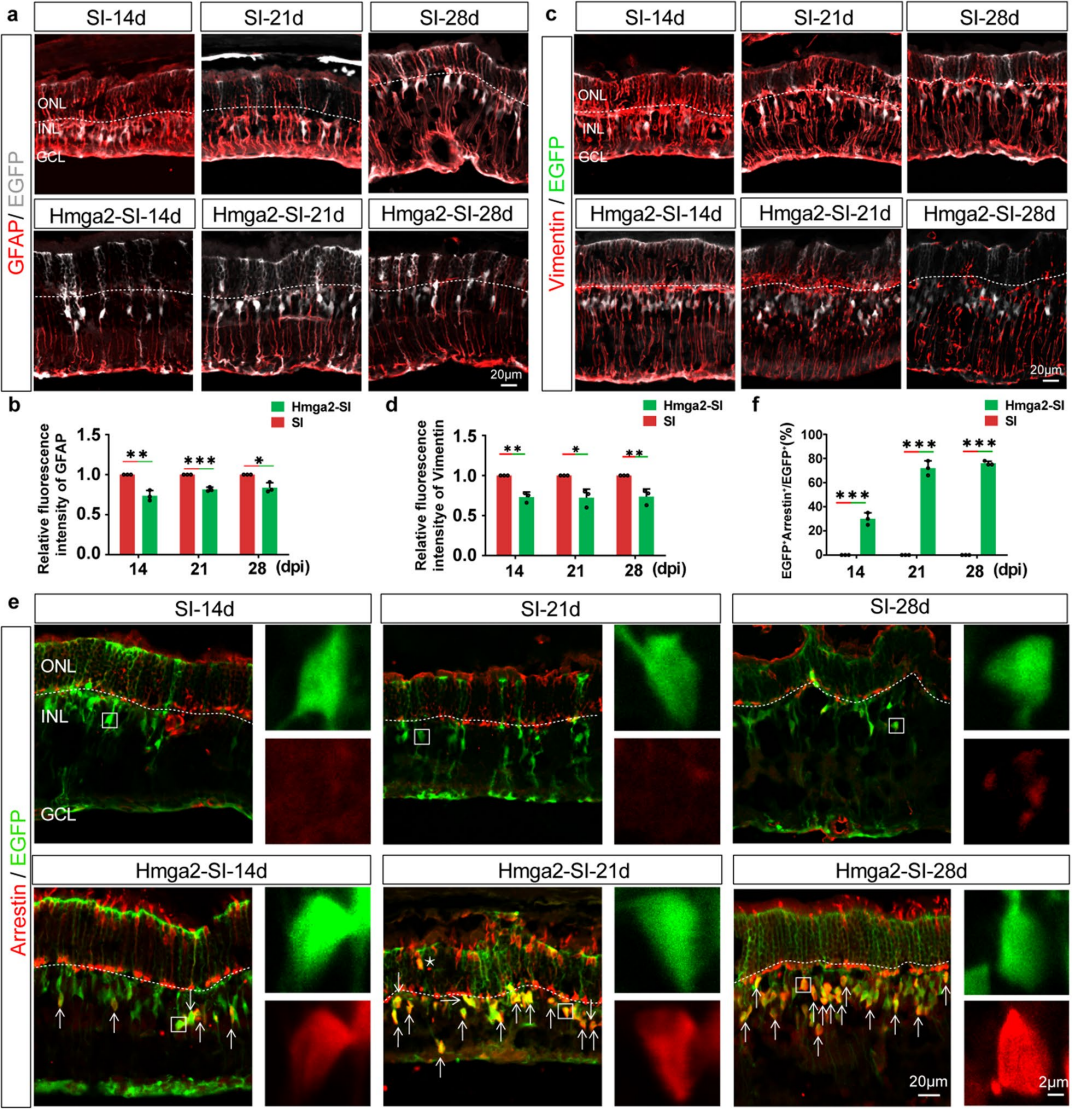
Hmga2 overexpressing in MG reduced MG gliosis and promoted MG to obtain cone’s marker. **a** Immunohistochemical labeling of GFAP and EGFP in the central retina of SI-treated and Hmga2-SI-treated groups at various time points. **b** Relative fluorescent intensity of GFAP protein in SI-treated and Hmga2-SI-treated retinas over time (*n* = 3). **c** Immunohistochemical labeling of Vimentin and EGFP in the central retina of SI-treated and Hmga2-SI-treated retinas at various time points. **d** Relative fluorescent intensity of Vimentin protein in SI-treated and Hmga2-SI-treated retinas over time (*n* = 3). **e** Immunohistochemical co-labeling of arrestin and EGFP in the central retina of SI-treated and Hmga2-SI-treated groups at various time points. Insets display the expression of each fluorescent reporter (White Square). White arrowheads identify the arrestin and EGFP double positive cells. Star marks identify the arrestin and EGFP double positive cells in ONL. **f** Ratio of arrestin and EGFP double positive MG in the retinas with SI treatment and Hmga2-SI treatment (*n* = 3). *ONL* Outer nuclear layer, *INL* Inner nuclear layer, *GCL* Ganglion cell layer. *P < 0.05; **P < 0.01; ***P < 0.001, Unpaired t-test was used in (**b**, **d**, **f**)

To verify the results of transcriptome, we tested the MG gliosis changes by GFAP and Vimentin staining, and discovered that Hmga2 overexpression could significantly reduce the expression of GFAP and Vimentin by roughly 30% in the MG of SI-injured mouse. The majority of the GFAP positive intermediate filaments were confined to inner nuclear layer (INL), and basal and apical MG processes seemed thinner in comparison to retinas infected with the control AAV, indicating that sustained overexpression of Hmga2 overexpression reduced MG gliosis (Fig. 6a–d).

Immunohistochemical staining of arrestin showed an enormous and significant rise in the proportion of arrestin positive MG in Hmga2 overexpressed retinas compared with that in the controls in all observed time points. There was no co-labeling of EGFP positive MG and arrestin in controls (SI-14d, SI-21d, SI-28d). However, about 30% EGFP positive MG were co-labeled with arrestin in HM-SI-14d group, and about 70% EGFP positive MG were co-labeled with arrestin in HM-SI-21d group and HM-SI-28d group (Fig. 6e, f). Notably, a few of the arrestin positive MG migrated into ONL, which had a round morphology without apparent cell processes, and did not get cone morphology (Additional file 1: Fig. S6). Meanwhile, arrestin positive cone cells increased concomitantly with the increased number of Hmga2-overexpressed MG, and the morphology of arrestin positive cone cells is better in Hmga2-overexpressed groups (Fig. 6e). These results revealed the possible quantitative correlation of arrestin positive cones with Hmga2-overexpressed MG.

MG reprogramming in the zebrafish retina was known to occur via dedifferentiation into a proliferative progenitor state [51], which was also observed in mammalian MG reprogrammed [15, 16, 20, 52]. Furthermore, as mentioned earlier, MG proliferation may be an indicator of glial scar formation, we thus wondered whether Hmga2-mediated reprogramming would promote or inhibit MG proliferation.

To test this, we labeled the cells with the proliferation markers, including Ccnd1, Ki67 and EdU. Ccnd1 is a G1-phase cell-cycle regulator, which oscillates minimally throughout the cell cycle [53]. Ccnd1 positive MG was observed in both Hmga2-overexpressed retinas and controls, but there were no significant differences among these groups at all observed time points (Additional file 1: Fig. S7a, b). Ki67 labels both interphase and mitotic cells, it is highly expressed in cycling cells while strongly down-regulated in resting G0 cells [54, 55]. However, there was no significant difference in the number of Ki67 positive cells in Hmga2-overexpessed retinas compared with control groups (Additional file 1: Fig. S7c). We also injected EdU into the AAV-infected mice to label S phase MG in two different methods, that is, EdU was administered three times (24 h, 12 h and 4 h before killing), or EdU was administered once a day from 1 to 14 dpi, spanning the early and late stages of SI injury (Additional file 1: Fig. S7d). Unfortunately, regardless of the method used, we did not observe significant difference in the EdU labeled MG in the retina between Hmga2 overexpressed and control retinas (Additional file 1: Fig. S7e, f).

Thus, Hmga2 overexpression in MG suppressed the expression of retinal gliosis-related protein (GFAP and Vimentin), while it failed to influence the MG proliferation and migration. Besides, overexpression of Hmga2 in MG promoted the expression of cone markers and enhanced survival of cones.

### Neuroprotective effects of Hmga2 on retina during SI-induced retinal injury

**Fig. 7:**
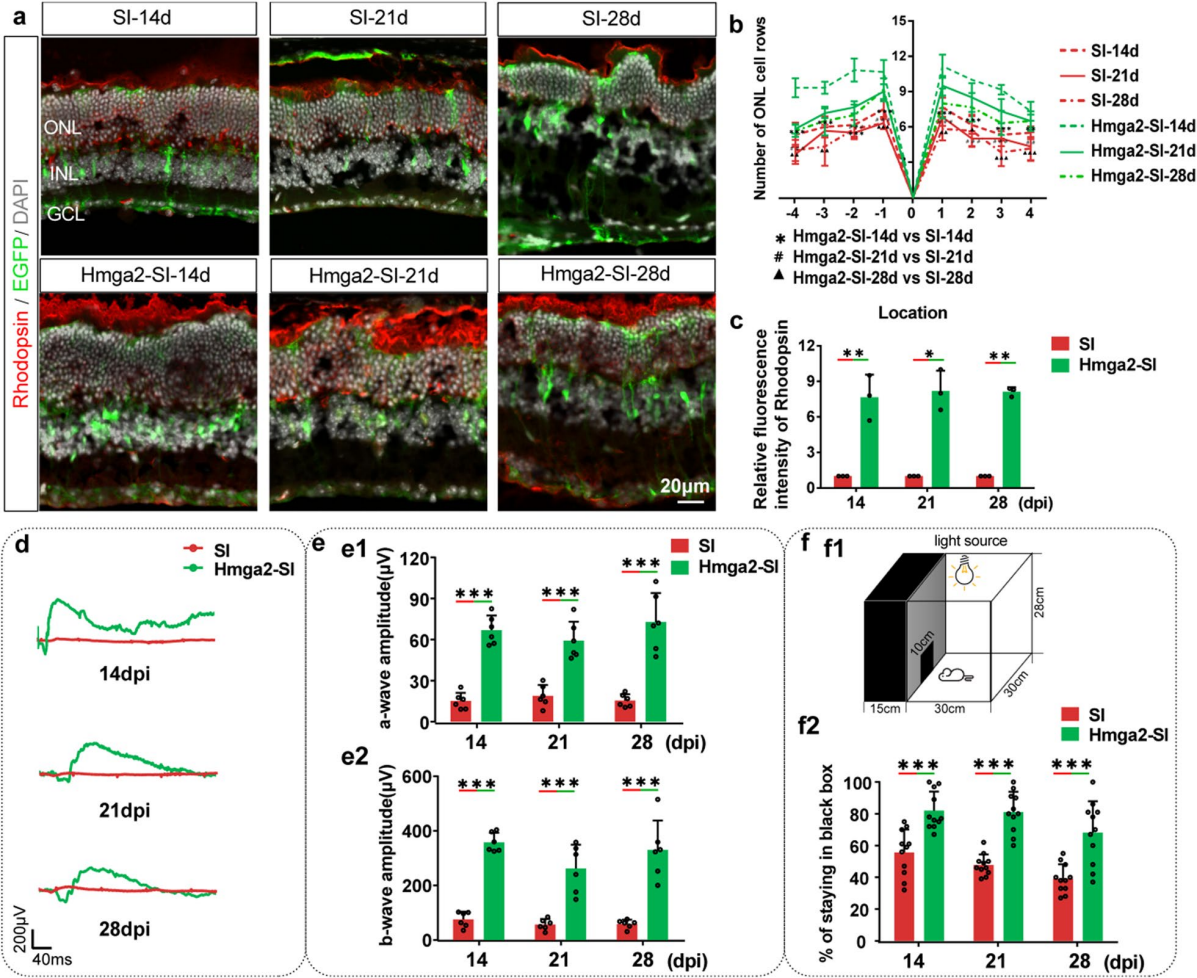
Neuroprotective effects of Hmga2 on retina during SI-induced retinal injury. **a** A comparison of retinal structures between SI-treated and Hmga2-SI-treated groups using DAPI and Rhodopsin staining. **b** The mean number of photoreceptor rows along central sections of the retina between SI-treated and Hmga2-SI-treated retinas at various time points (*n* = 3). **c** Relative fluorescent intensity of Rhodopsin protein in the central retinas with SI treatment and Hmga2-SI treatment at various time points (*n* = 3). **d** ERG waveforms in scotopic conditions between SI-treated and Hmga2-SI-treated retinas at various time points. **e** A comparison of the a-wave (e1) and b-wave (e2) amplitudes in scotopic conditions between the SI-treated and Hmga2-SI-treated retinas at various time points (*n* = 6). **f** The light/dark transition test sketch map (f1) and a comparison of the duration time proportion in the black box between the SI-treated and Hmga2-SI-treated retinas at various time points (f2) (*n* = 11). *ONL* Outer nuclear layer, *INL* Inner nuclear layer, *GCL* Ganglion cell layer. *P < 0.05; **P < 0.01; ***P < 0.001; #* P* < 0.05; # #* P* < 0.01; ###* P* < 0.001; ▲*P* < 0.05; ▲▲* P* < 0.01,;▲▲▲* P* < 0.001. Unpaired *t*-test was used in (**b**, **c**, **e**, **f**)

Except that Hmga2 overexpression in MG promotes cone survival, Hmga2-treated mice showed significantly thicker ONL compared with control AAV-infected injury groups (Fig. 5c, d). Meanwhile, Rhodopsin, which is the most abundant protein in the rod cells was also up-regulated by Hmga2 treatment (Fig. 7a–c) [56]. Moreover, Hmga2 overexpression in MG significantly enhanced a-wave and b-wave amplitudes by compared with the control AAV-infected SI injured mice (Fig. 7d, e). The dark-adapted a-wave is generated by the hyperpolarization of the outer segments of rods and cones in response to light stimulation, and the origin of dark-adapted b-wave arises in either bipolar cells, MG cells, or both [57]. Thus, the improvement of amplitude of a- and b-wave confirmed the neuroprotective effect of Hmga2 on photoreceptors. In addition, black-and-white test shown SI-injured mice treated with experimental AAV significantly lengthened their stay in the dark zone at each time point (*P* < 0.05) (Fig. 7f).

Taken together, these results indicated that Hmga2 was successful in halting the loss of photoreceptors, and had significant protective effects on mouse vision after retinal injury. Since 14dpi is a late stage of SI injury, and there were no significant differences in ERG and light/dark test results between experimental-AAV-infected groups. We speculated that the protective effect of the Hmga2 could last for a relatively long time.

## Discussion

A specific time window (3 dpi-5 dpi) for MG gliosis fate determination was identified firstly by GFAP, proliferation, migration and transcriptional examination during the acute retinal injury. Panels of genes promoting MG gliosis and proliferation were activated transiently at 3dpi followed by a gradual reversion to a resting state (7 dpi-14 dpi). Previous report showed that 1 day following the experimental retinal detachment, GFAP expression in MG cells was significantly increased. At 3 dpi, cell hypertrophy of MG is apparent in the retina and subretinal region, which usually caused subretinal fibrosis [58, 59]. Blackshaw et al. reported that mouse MG showed rapid transcriptional changes after NMDA or light injury as early as 3 h after injury and then returned to a resting-like state in 36–48 h [22]. In view of this, the time of MG activation may be determined by the types of injury models. Injury models differed in terms of the damage types, the cell types affected, and the intensity of the MG-mediated response [60].

We failed to screen out the satisfying candidate DEGs for MG reprogramming using scRNA seq, partially due to the low MG capture ratio (about 500 MG per sample). The ideal approach is to use flow cytometry to sort MG cells from multiple retina and then perform scRNA seq. Besides, only about 5% of MG expressed Hmga2 at 3 dpi, suggesting the combined analysis of multi-omics was necessarily applied to identify lowly expressed but critical genes. Importantly, although our screening assay was an appropriate approach to identify reprogramming factors, it does not exclude other more reasonable strategies for screening MG candidate factors. In addition, Hmga2 has a higher priority than other candidate genes, which did not mean that other candidates were unable to reprogram MG, since we did not validate the expression of these factors.

In our study, the inhibitory effect of Hmga2 on retinal gliosis and the promotion of photoreceptor cell survival were preliminarily determined through bulk-RNA seq. Through immunohistochemical staining, it was further confirmed that Hmga2 can inhibit MG cell gliosis and enable MG to obtain the typical marker of cone cells, arrestin. A small amount of MG with “new identity” also migrated to ONL. In addition, overexpression of Hmga2 in MG improved the survival of cone and rod cells, effectively protecting photoreceptors from loss and maintaining vision.

Currently, reprogramming MG into cone cells is a world-class challenge. One reports reported that co-expressed Ikzf1 and Ikzf4 in MG, resulting in a small number of MG cells obtaining cone cell markers Rxrg. However, other cone cell markers such as S-opsin, L/M-opsin, arrestin, and Peanut agglutinin were not detected, indicating incomplete transdifferentiation of MG into cone cells [18]. Compared with this report, Hmga2 could achieve efficient promotion of MG to obtain markers of cone cells.

Moreover, only a few studies have shown that MG reprogramming effectively improves visual function. Yang et al. used RNA targeting of the CRISPR system to induce the conversion of MG cells to RGC and achieve partial recovery of visual response [21]. Similarly, Xiang et al. confirmed that RGC derived from MG could improve retinal function [61]. Chen et al. reported that MG derived rod cells could be integrated into the retinal circuit and restore visual function in Gnat1^rd17^Gnat2^cpfl3^ mice [16]. Some studies only conducted electrical activity detection on reprogrammed neurons, and did not demonstrate their recovery effect on visual function [15, 19, 62]. In our study, Hmga2 showed a significant improvement in visual function, but Hmga2 and arrestin positive MG rarely migrated to ONL to replace lost neurons. Therefore, the underlying mechanisms of MG with new phenotype effectively protected the injured photoreceptors from cell loss require further investigation.

Hmga2 protein is a small nonhistone nuclear protein that can bind to DNA in small grooves and alter chromatin conformation and accessibility through several regulatory factors involved in regulating gene expression [49]. Ahmad et al. demonstrated that inhibiting the expression of Hmga2 could achieve the positive effect of let-7 on MG differentiation [63]. Patterson et al. provided evidences that the let-7/Hmga2/Hes5 (a Notch effector) pathway could regulate the switch from neurogenesis to gliogenesis in human neural stem cells (NSCs). In our study, the overexpression of Hes5 clearly increased in HM-SI-14d group compared with SI-14d group (Fig. 6c1) (Additional file 1: Table S4), but it is unlikely to be caused by the activation of Notch expression [64]. In other hand, Hmga2 promotes the expression of insulin-like growth factor 2 mRNA-binding protein 2 (Igf2bp2) in neocortical neural precursor cells (NPCs), and overexpression of Igf2bp2 increased the neurogenic potential and suppressed astrocytic differentiation of late-stage NPCs [65]. According to the sequencing data, the expression of Igf2bp2 was up-regulated in both HM-SI-14d and HM-SI-28d groups compared with SI-14d and SI-28d groups, respectively (Additional file 1: Table S4). It revealed that the glial/neurogenic switch via Hmga2 might through the up-regulation of Hes5 and Igf2bp2 expression in the retina.

Hmga2 has been found in adult stem cells and NSCs [66]. Overexpression of Hmga2 induced human somatic cells to reprogram into NSCs and increased hematopoietic stem cell self-renewal in mice [42–46]. Hmga2 also aided tumorigenesis by promoting cell cycle entry [41]. Our results showed that overexpression of Hmga2 in MG did not promote MG cell cycle reentry by immunohistochemical staining. Meanwhile, bulk-RNA seq analysis did not reveal the up-regulation of proliferation-related genes or pathways following overexpression of Hmga2. These results suggested that mouse MG reprogramming via Hmga2 overexpression did not reprogram through a proliferating progenitor-like state and instead were consistent with direct reprogramming. It was concordant with works in mice showing some MG reprogramming to neuron-like cells without proliferation [17, 18, 21, 61]. Hmga2 overexpression alone could not promote MG to proliferate, indicating other factors might be needed.

Using the string database, we generated a protein–protein interaction circuit of *Hmga2*/*Ccnd1*/*Vimentin*/*Gfap*/*Rhodopsin*/*Arrestin*/*Gnas*. Gnas activates adenylyl cyclase in the signal transduction pathway of G protein-coupled receptor, leading to the increase of cAMP level and participating in the regulation of cell growth and cell division. Meanwhile, *Gnas* was also down-regulated in HM-SI-28d group compared with SI-28d group (Additional file 1: Table S4). It suggested that *Gnas* might be a hub gene for the effects of Hmga2 on MG.

Taken together, the functions of Hmga2 on MG gliosis alleviation, glial/neurogenic switch and vision preservation might be controlled by multiple pathways. Certainly, it did not exclude that Hmga2 regulated the expression of photoreceptor survival related genes and gliosis related genes directly which contributed to the vision preservation. A more ideal way is to apply ATAC seq to further elucidate the mechanism of Hmga2 on MG fate switch.

To better understand the effect of Hmga2 on cell fate determination of activated MG and the neuroprotection on SI-induced retinal injury, especially knockdown of *Hmga2* in MG might be necessary. However, in the retina of healthy adult mice, Hmga2 protein was not detected in MG. Five days post SI-induced retina injury, the level of Hmga2 protein reached the peak point of the experiment stage, while it was less than 10% (Fig. 4g). Cre mice carrying MG-specific promoters and loxp mice carrying Hmga2 genes might be ideal tools for achieving gene knockout. SI-induced retinal injury is one of the acute retinal injury mouse models, transgenic mice models for RP or AMD mice models might be used to verify the neuroprotection function of Hmga2 on the degenerative retina. To confirm the existence of MG cell subpopulations (cluster1 and cluster3) in healthy retina, it is necessary to identify them with more specific cell markers.

## Conclusions

In the present study, we established a rational approach to determining the critical time window of MG gliosis and screening regulators for MG reprogramming process after acute injury. Using bulk-RNA seq and scRNA seq, *Hmga2* was screened out and identified as an essential epigenetic regulator. Overexpressing of Hmga2 switched the MG cell fate from gliosis to obtain cone’s marker and restored visual function partially.

For clinical applications, the ideal reprogramming strategy is to reprogram MG into other neurons in a way similar to zebrafish MG reprogramming. However, the majority of current strategies reprogram MG into other retinal neurons without proliferation, which will lead to the reduction of MG numbers. Hmga2-mediated MG cell fate switch did not require MG to transdifferentiate into neurons completely for visual function repair, thereby avoided an important depletion of the MG population. It might be a novel therapy for RP or AMD.

### Supplementary Information

**Additional file 1: Figure S1.** Sodium iodate induced acute retinal injury in MG lineage tracing mice. **a** Experimental design for SI treatment and multiple analysis time points in MG lineage tracing mice. **b** MG were marked with tdTomato red fluorescence in MG lineage tracing mice. A whole retinal cross section and a slightly expanded view are displayed (inner side). **c** The nucleus of tdTomato positive cells are co-labeled with the MG marker Sox9 (c1), not co-labeled with the ganglion cell marker-Rbpms (c2), photoreceptor marker-Recoverin (c3), and cone marker-arrestin (c4). **d** Ratio of Sox9, Rbpms, Recoverin and arrestin co-labeling with tdTomato cells in each retina section (*n* = 3). **e** A comparison of retinal structures between control and SI-treated groups using DAPI staining. **f** The mean number of photoreceptor rows along central sections of the retina between control and SI-treated retinas (*n* = 3). **g** ERG waveforms in scotopic condition between control and SI-treated retinas. **h** A comparison of a-wave (h1) and b-wave (h2) amplitudes in scotopic conditions between control and SI-treated retinas (*n* = 5). *ONL* Outer nuclear layer, *INL* Inner nuclear layer, *GCL* Ganglion cell layer. *P < 0.05; **P < 0.01; ***P < 0.001. one-way ANOVA test was used in (**d**, **f**, **h**). **Figure S2.** The most obvious transcriptional alterations of Müller glia occurred at 3 days post injection. **a** A feature expression dot map of the principal retinal class markers over 10 clusters of retinal cells. **b** UMAP image of 54,236 retinal cells colored by annotation of 10 transcriptionally different clusters. **c** The number of DEGs in the SI-3d and SI-7d groups compared with control in MG. **d** Comparing representative DEGs of MG from the SI-3d group compared with the control group in MG. **e** Enriched GO terms of DEGs in the SI-3d group compared with the control group in MG. **Figure S3.** Potential genes for MG reprogramming process among DEGs from bulk-RNA seq and scRNA seq. **a** UniProt database annotations for keywords of module 1. Line thickness represents the degree of data support; a minimum interaction score of 0.5 is needed. **b** Protein-protein interaction network of screened 10 DEGs of scRNA seq from Venn diagram. **c** Relative expression of screened 10 DEGs from scRNA seq in each group, the colors correspond to relative expression. All 10 DEGs had |log2 (fold changes)| values between 1 and 2 at 3 dpi. **Figure S4.** Transcriptional expression of Hmga2 and other related genes in control and SI-injury groups. **a** RT-qPCR analysis of Hmga2, Igfbp3, Tgfb1, and Ccnd1 transcription levels compared with bulk-RNA seq (*n* = 3). **b** A feature dot map of Hmga2 average expression and percent expression in 10 clusters of retinal cells from the control, SI-3d and SI-7d groups. *P < 0.05; **P < 0.01; ***P < 0.001. One-way ANOVA test was used in (**a**). **Figure S5.** Co-labeling of EGFP with Sox9 and Hmga2 protein in control and experimental AAVs. **a** Experimental design for Hmga2 overexpression by AAV prior to SI treatment, with multiple time points for analysis in mice. **b** Virus-infected MG are labeled with EGFP green fluorescence in the uninjured retina, and an entire retinal cross section is shown. **c** Immunohistochemical co-labeling of Sox9 and EGFP protein in control and experimental AAV-infected retinas. White hollow arrows identify EGFP positive and Sox9 negative cells. **d** Ratio of Sox9 and EGFP double positive cells in control and experimental AAV-infected retinal sections (*n* = 5). **e** Immunohistochemical co-labeling of Hmga2 and EGFP protein in control and experimental AAV-infected retinas. **f** Ratio of Hmga2 and EGFP double positive cells in control and experimental AAV-infected retinal sections (*n* = 5). *ONL*, outer nuclear layer,* INL*, inner nuclear layer, *GCL*, ganglion cell layer, *P < 0.05; **P < 0.01; ***P < 0.001, Unpaired t-test was used in (**d**, **f**). **Figure S6.** The morphology of arrestin and EGFP double positive cells in ONL. **a** Representative images of arrestin and EGFP double positive cells in ONL at Hmga2-SI treated group. Insets display the expression of each fluorescent reporter (White Square). *ONL* Outer nuclear layer, *INL* Inner nuclear layer, *GCL* Ganglion cell layer. **Figure S7.** Hmga2 expression in MG did not promote MG proliferation **a** Immunohistochemical co-labeling of EGFP and Ccnd1 protein in the central retina that received SI treatment and Hmga2-SI treatment at various time points. White arrowheads identify where the MG is Ccnd1 positive. Insets display the expression of each fluorescent reporter (White Square). **b** Ratio of Ccnd1 positive MG in each section of the retina with SI treatment and Hmga2-SI treatment at various time points (*n* = 3). **c** Ratio of Ki67 positive MG in each section of the retina with SI treatment and Hmga2-SI treatment at various time points (*n* = 3). **d** Two EdU injection schemes during Hmga2 overexpression and SI treatment, and multiple time points for analysis in mice. **e** Ratio of EdU positive MG labeled by the first method in each section of the retinas with SI treatment and Hmga2-SI treatment at various time points (*n* = 3). **f** Ratio of EdU positive MG labeled by the second method in each section of the retinas in the retinas with SI treatment and Hmga2-SI treatment at various time points (*n* = 3). *ONL*, outer nuclear layer, *INL*, inner nuclear layer, *GCL*, ganglion cell layer, *P < 0.05; **P < 0.01; ***P < 0.001, Unpaired *t*-test was used in (**b**, **c**, **e**, **f**). **Figure S8.** Full-length gels and blot of Fig. 1c. **a** Full-length gels and blot of GFAP in control, SI-1d, SI-3d, SI-5d and SI-14d groups. The green box represents the cropping position. **b** Full-length gels and blot of β-actin in control, SI-1d, SI-3d, SI-5d and SI-14d groups. The red box represents the cropping position. **Table S1.** Primary and secondary antibodies used in immunohistochemistry. **Table S2.** Primary and secondary antibodies used in western blot. **Table S3.** Primer sequences for RT-qPCR analysis. **Table S4.** Comparison of the transcriptional expression of Hmga2 and other related genes in the retina with SI treatment and Hmga2-SI treatment.

## Data Availability

All bulk-RNA sequencing and scRNA sequencing data generated or analyzed during this study are included in this article. The original raw sequence data have been deposited in the Genome Sequence Archive (Genomics, Proteomics & Bioinformatics 2021) in National Genomics Data Center (Nucleic Acids Res 2022), China National Center for Bioinformation/Beijing Institute of Genomics, Chinese Academy of Sciences (GSA: CRA010065) that are publicly accessible at https://bigd.big.ac.cn/gsa/browse/CRA010065

## Abbreviations

MG: Müller glia
RP: Retinitis pigmentosa
SI: Sodium iodate
scRNA seq: Single-cell RNA sequencing
AAV: Adeno-associated viruses
AMD: Age-related macular degeneration
RPE: Retinal pigment epithelium
RPC: Retinal progenitor cell
GFAP: Glial fibrillary acidic protein
Hmga2: High mobility histone A2
ONL: Outer nuclear layer thickness
ERG: Electroretinogram
PFA: Paraformaldehyde
DEGs: Differentially expressed genes
GO: Gene ontology
RT-PCR: Real-time fluorescence quantitative polymerase chain reaction
INL: Inner nuclear layer
GCL: Ganglion cell layer
Igf2bp2: Insulin-like growth factor 2 mRNA-binding protein 2
NSC: Neural stem cell
