# Supplementary material for: Identifying Hmga2 preserving visual function by promoting a shift of Müller glia cell fate in mice with acute retinal injury
Source: Stem Cell Res Ther. 2024 Feb 27;15:54. doi: 10.1186/s13287-024-03657-9 (PMC10900711; doi:10.1186/s13287-024-03657-9)
Supplement: Supplementary file 1 — Additional file 1: Figure S1. Sodium iodate induced acute retinal injury in MG lineage tracing mice. a Experimental design for SI treatment and multiple analysis time points in MG lineage tracing mice. b MG were marked with tdTomato red fluorescence in MG lineage tracing mice. A whole retinal cross section and a slightly expanded view are displayed (inner side). c The nucleus of tdTomato positive cells are co-labeled with the MG marker Sox9 (c1), not co-labeled with the ganglion cell marker-Rbpms (c2), photoreceptor marker-Recoverin (c3), and cone marker-arrestin (c4). d Ratio of Sox9, Rbpms, Recoverin and arrestin co-labeling with tdTomato cells in each retina section (n = 3). e A comparison of retinal structures between control and SI-treated groups using DAPI staining. f The mean number of photoreceptor rows along central sections of the retina between control and SI-treated retinas (n = 3). g ERG waveforms in scotopic condition between control and SI-treated retinas. h A comparison of a-wave (h1) and b-wave (h2) amplitudes in scotopic conditions between control and SI-treated retinas (n = 5). ONL Outer nuclear layer, INL Inner nuclear layer, GCL Ganglion cell layer. *P < 0.05; **P < 0.01; ***P < 0.001. one-way ANOVA test was used in (d, f, h). Figure S2. The most obvious transcriptional alterations of Müller glia occurred at 3 days post injection. a A feature expression dot map of the principal retinal class markers over 10 clusters of retinal cells. b UMAP image of 54,236 retinal cells colored by annotation of 10 transcriptionally different clusters. c The number of DEGs in the SI-3d and SI-7d groups compared with control in MG. d Comparing representative DEGs of MG from the SI-3d group compared with the control group in MG. e Enriched GO terms of DEGs in the SI-3d group compared with the control group in MG. Figure S3. Potential genes for MG reprogramming process among DEGs from bulk-RNA seq and scRNA seq. a UniProt database annotations for keywords of module [file 13287_2024_3657_MOESM1_ESM.docx]

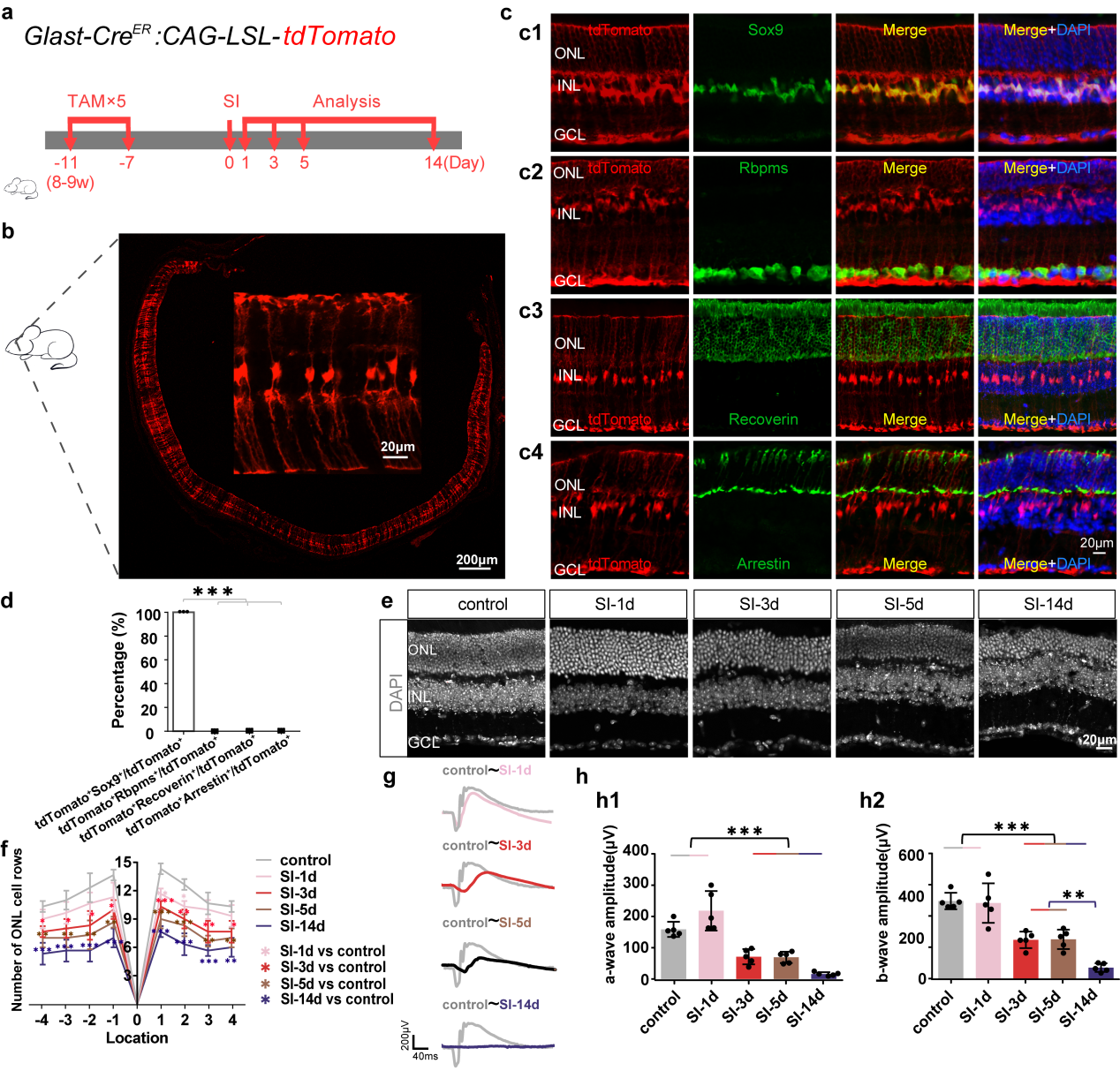


**Fig. S1 Sodium iodate induced acute retinal injury in MG lineage tracing mice.**

a) Experimental design for SI treatment and multiple analysis time points in MG lineage tracing mice.

b) MG were marked with tdTomato red fluorescence in MG lineage tracing mice. A whole retinal cross-section and a slightly expanded view are displayed (inner side).

c) The nucleus of tdTomato positive cells are co-labeled with the MG marker Sox9 (c1), not co-labeled with the ganglion cell marker-Rbpms (c2), phoreceptor marker-Recoverin (c3), and cone marker-Arrestin (c4).

d) Ratio of Sox9, Rbpms, Recoverin and Arrestin co-labeling with tdTomato cells in each retina section (n=3).

e) A comparison of retinal structures between control and SI-treated groups using DAPI staining.

f) The mean number of photoreceptor rows along central sections of the retina between control and SI-treated retinas (n=3).

g) ERG waveforms in scotopic condition between control and SI-treated retinas.

h) A comparison of a-wave (h1) and b-wave (h2) amplitudes in scotopic conditions between control and SI-treated retinas (n=5).

ONL, outer nuclear layer; INL, inner nuclear layer; GCL, ganglion cell layer; **P* < 0.05, ***P* < 0.01, ****P* < 0.001, One-way ANOVA test was used in fig d, f, h.


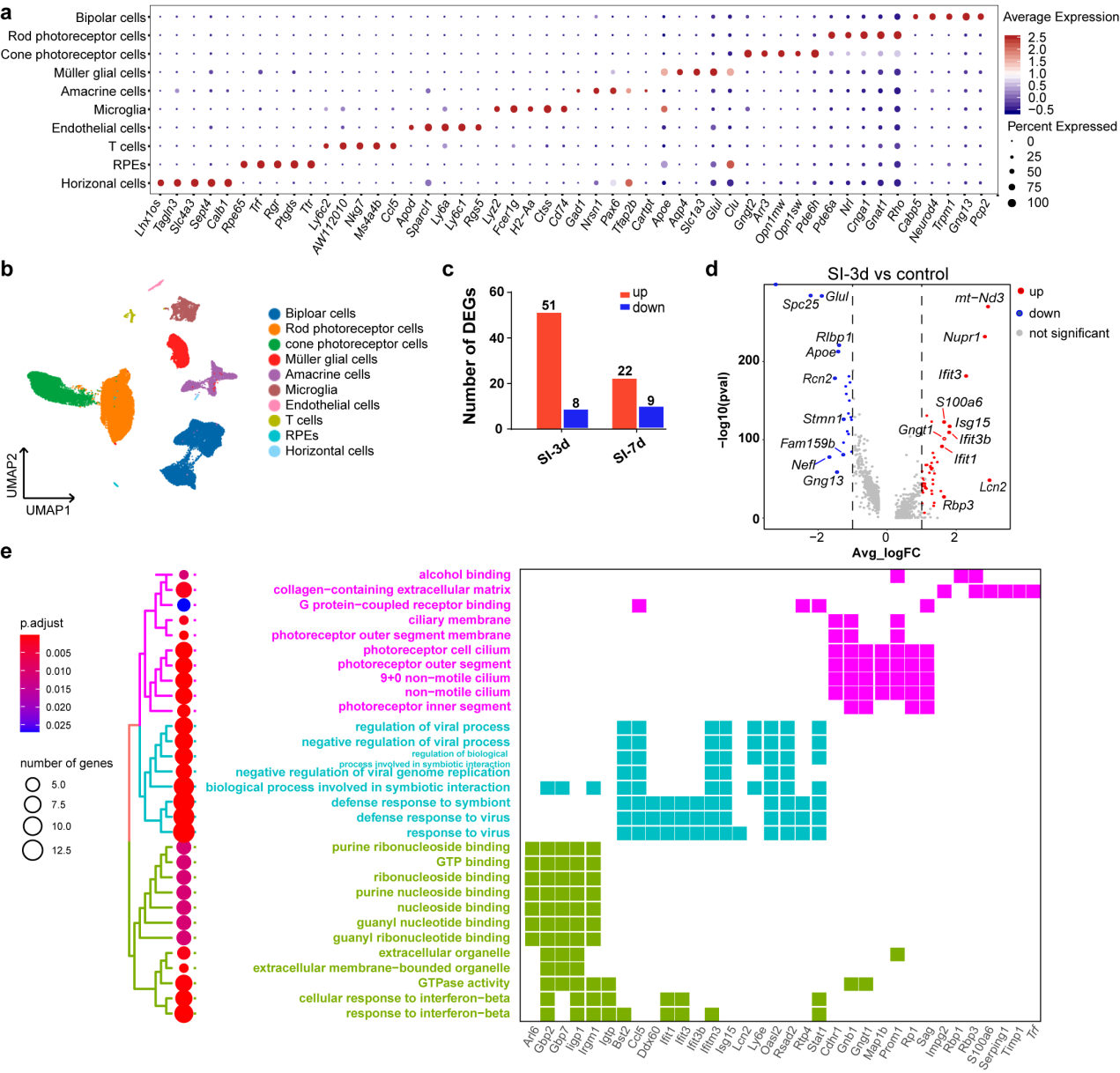


**Fig. S2 The most obvious transcriptional alterations of Müller glia occurrence occured at 3 days post-injection.**

a) A feature expression dot map of the principal retinal class markers over 10 clusters of retinal cells.

b) UMAP image of 54,236 retinal cells colored by annotation of 10 transcriptionally different clusters.

c) The number of DEGs in the SI-3d and SI-7d groups compared with control in MG.

d) Comparing representative DEGs of MG from the SI-3d group compared with the control group in MG.

e) Enriched GO terms of DEGs in the SI-3d group compared with the control group in MG.


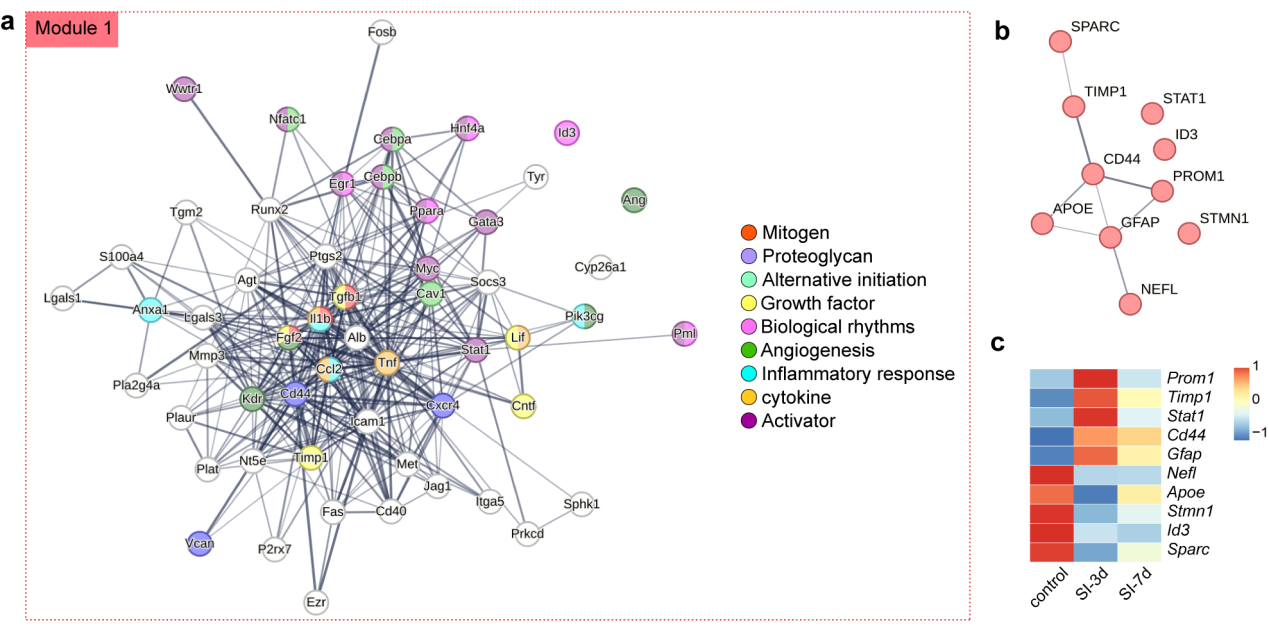


**Fig. S3 Potential genes for MG reprogramming process among DEGs from bulk-RNA-seq and scRNA-seq..**

a) UniProt database annotations for keywords of module 1. Line thickness represents the degree of data support; a minimum interaction score of 0.5 is needed.

b) Protein–protein interaction network of screened 10 DEGs of scRNA-seq from Venn diagram.

c) Relative expression of screened 10 DEGs from scRNA-seq in each group, the colors correspond to relative expression. All 10 DEGs had |log_2_ (fold changes)| values between 1 and 2 at 3 dpi.

**
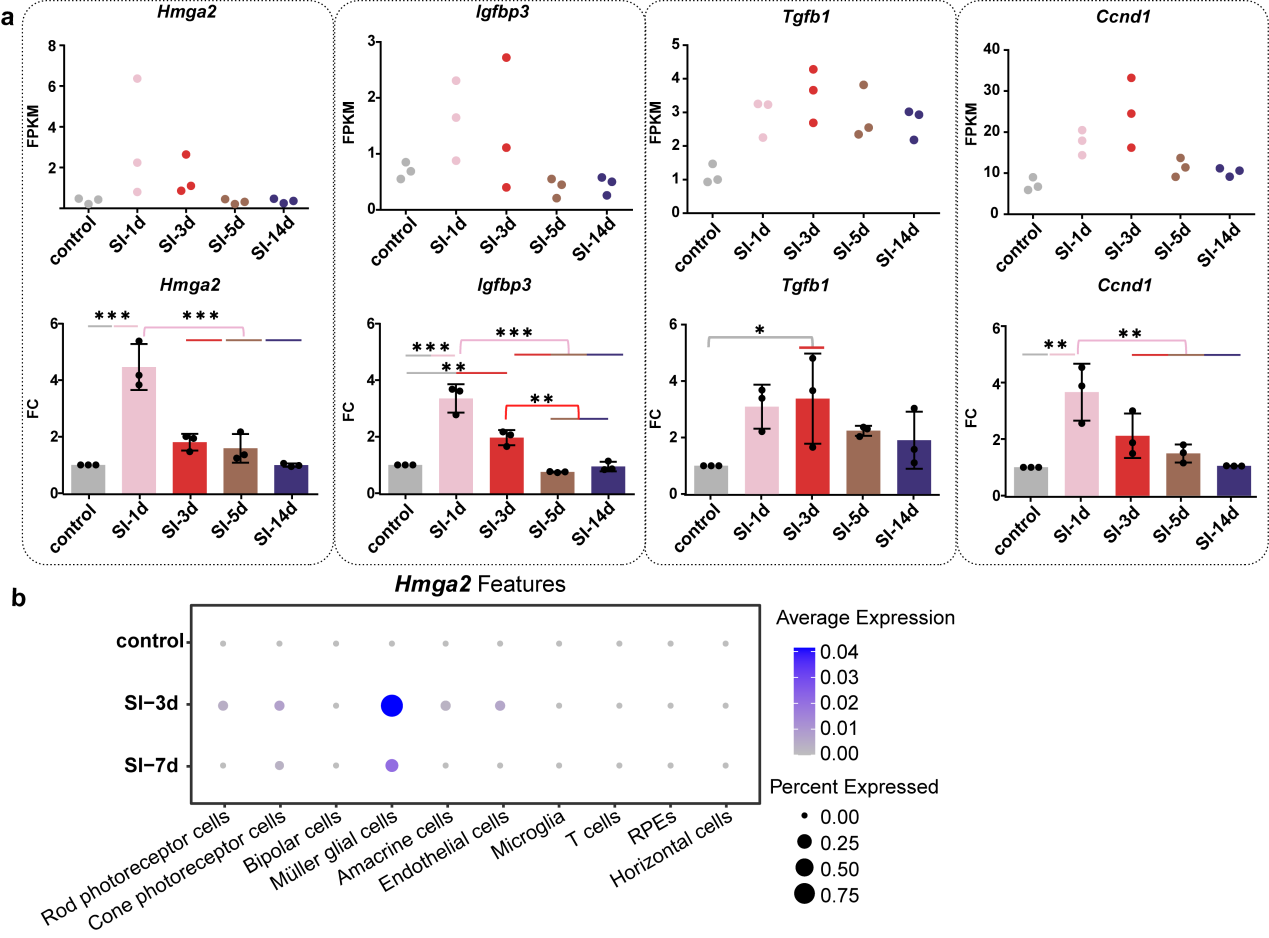
**

**Fig. S4 Transcriptional expression of *Hmga2* and other related genes in control and SI-injury groups.**

a) RT-qPCR analysis of *Hmga2, Igfbp3, Tgfb1,* and *Ccnd1* transcription levels compared with bulk-RNA seq (n=3).

b) A feature dot map of *Hmga2* average expression and percent expression in 10 clusters of retinal cells from the control, SI-3d and SI-7d groups.

**P* < 0.05, ***P* < 0.01, ****P* < 0.001, One-way ANOVA test was used in fig a.


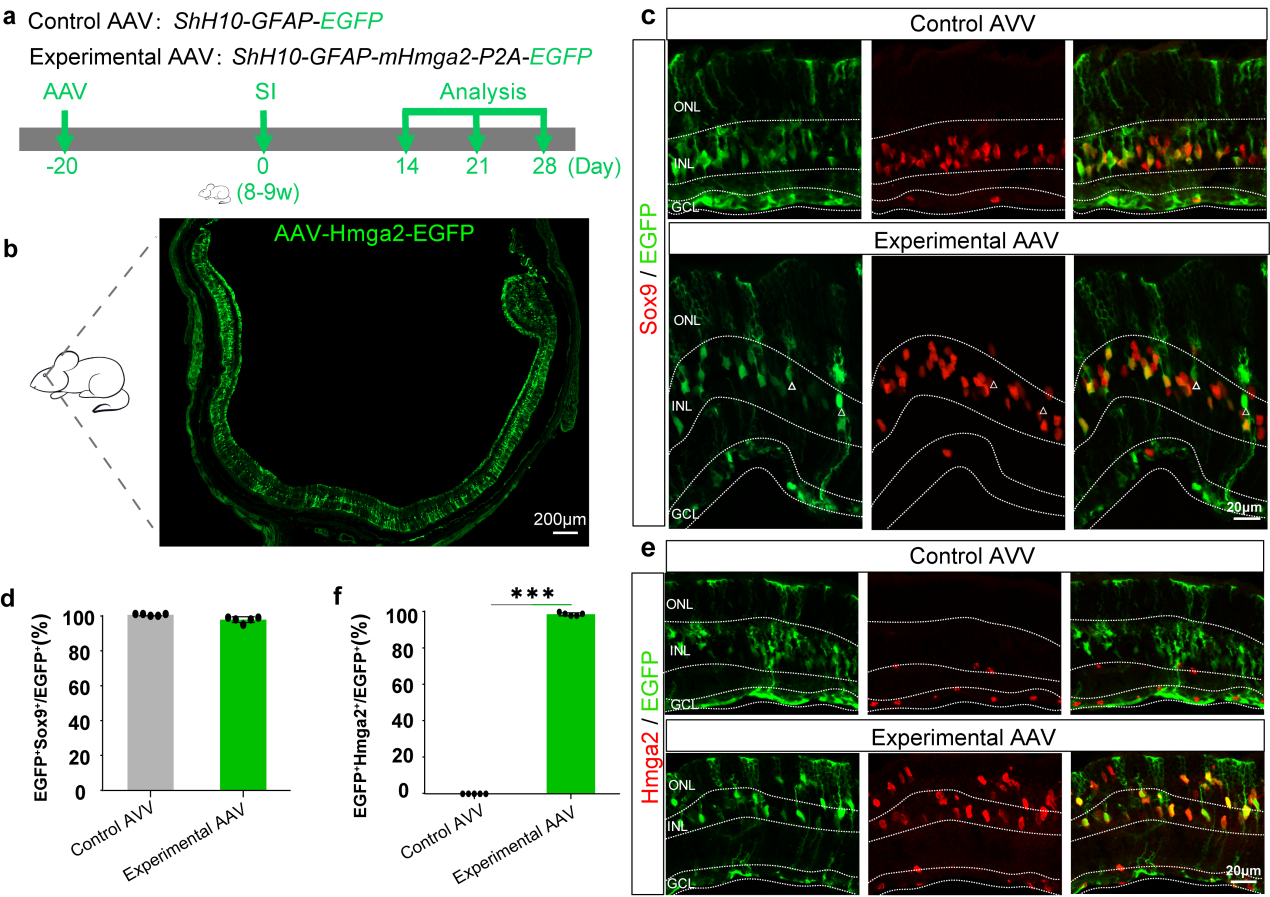


**Fig. S5 Co-labeling of EGFP with Sox9 and Hmga2 protein in control and experimental AAVs**

a) Experimental design for Hmga2 overexpression by AAV prior to SI treatment, with multiple time points for analysis in mice.

b) Virus-infected MG are labeled with EGFP green fluorescence in the uninjured retina, and an entire retinal cross-section is shown.

c) Immunohistochemical co-labeling of Sox9 and EGFP protein in control and experimental AAV-infected retinas. White hollow arrows identify EGFP-positive and Sox9 negative cells**.**

d) Ratio of Sox9 and EGFP double positive cells in control and experimental AAV-infected retinal sections (n=5).

e) Immunohistochemical co-labeling of Hmga2 and EGFP protein in control and experimental AAV-infected retinas.

f) Ratio of Hmga2 and EGFP double positive cells in control and experimental AAV-infected retinal sections (n=5).

**P* < 0.05, ***P* < 0.01, ****P* < 0.001, Unpaired t-test was used in fig d, f.


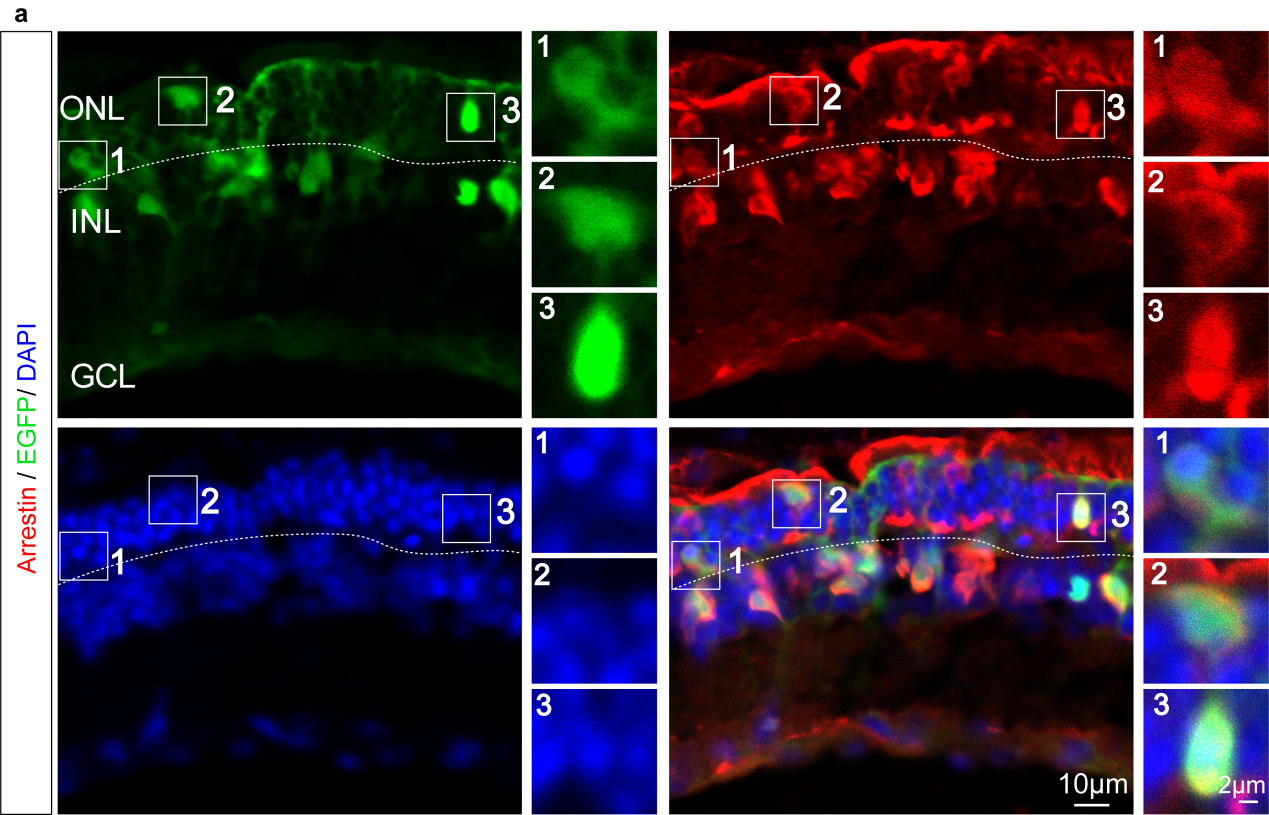


**Fig. S6** The morphology of Arrestin and EGFP double-positive cells in ONL

a) Representative images of Arrestin and EGFP double-positive cells in ONL at Hmga2-SI treated group. Insets display the expression of each fluorescent reporter (White Square).

ONL, outer nuclear layer; INL, inner nuclear layer; GCL, ganglion cell layer


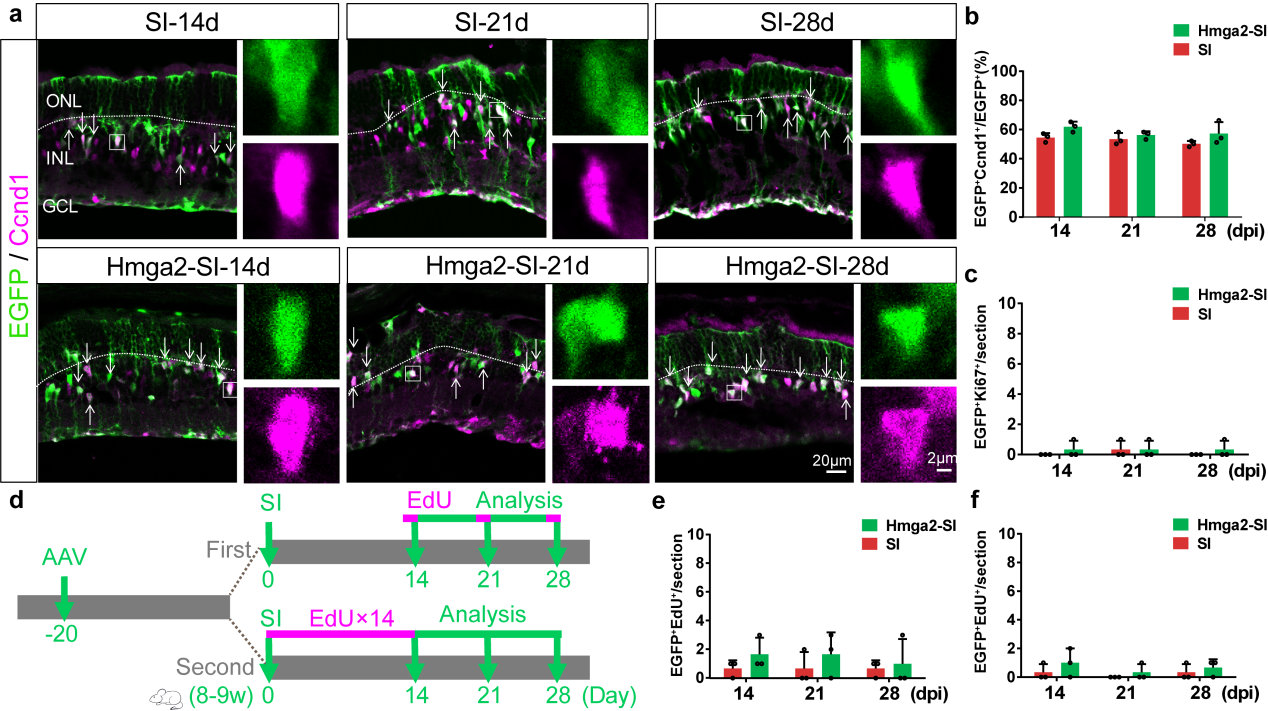


**Fig. S7 Hmga2 expression in MG did not promote MG proliferation**

a) Immunohistochemical co-labeling of EGFP and Ccnd1 protein in the central retina that received SI treatment and Hmga2-SI treatment at various time points. White arrowheads identify where the MG is Ccnd1 positive. Insets display the expression of each fluorescent reporter (White Square).

b) Ratio of Ccnd1-positive MG in each section of the retina with SI treatment and Hmga2-SI treatment at various time points (n=3).

c) Ratio of Ki67-positive MG in each section of the retina with SI treatment and Hmga2-SI treatment at various time points (n=3).

d) Two EdU injection schemes during Hmga2 overexpression and SI treatment, and multiple time points for analysis in mice.

e) Ratio of EdU-positive MG labeled by the first method in each section of the retinas with SI treatment and Hmga2-SI treatment at various time points (n=3).

f) Ratio of EdU-positive MG labeled by the second method in each section of the retinas in the retinas with SI treatment and Hmga2-SI treatment at various time points (n=3).

**P* < 0.05, ***P* < 0.01, ****P* < 0.001, Unpaired t-test was used in fig b, c, e, f.


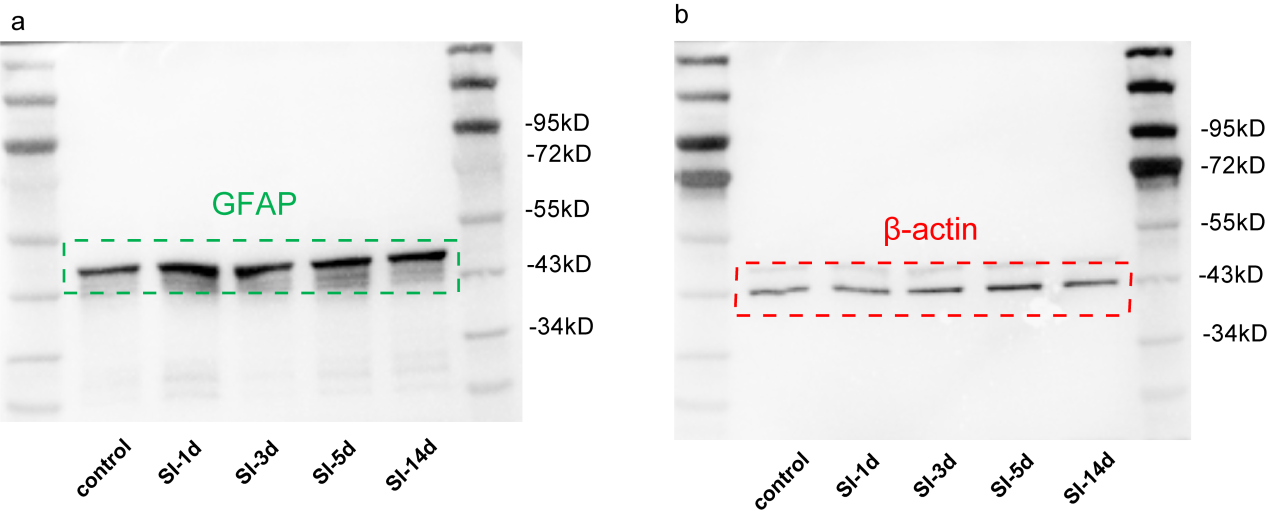


**Fig. S8 Full-length gels and blot of Figure 1c**

a) Full-length gels and blot of GFAP in control, SI-1d, SI-3d, SI-5d and SI-14d groups. The green box represents the cropping position.

b) Full-length gels and blot of β-actin in control, SI-1d, SI-3d, SI-5d and SI-14d groups. The red box represents the cropping position.

**Table S1.** **Primary and secondary antibodies used in immunohistochemistry**

| Antibodies | Manufacturer and lot | Dilution ratio |
| --- | --- | --- |
| Sox9 | Abcam ab185966 | 1:500 |
| Rbpms | Abcam ab152101 | 1:400 |
| Recoverin | Abcam ab31928 | 1:400 |
| Arrestin | Sigma ab15282 | 1:400 |
| GFAP | DAKO-Z033401 | 1:500 |
| Vimentin | ABclonal WH223727 | 1:400 |
| Hmga2 | CST-8179 | 1:400 |
| Rhodopsin | Abcam ab221664 | 1:400 |
| Ccnd1 | ABclonal19038 | 1:100 |
| Ki67 | Abcam Ab15580 | 1:400 |
| 488 donkey-mouse | Invitrogen A21202 | 1:500 |
| 568 donkey-rabbit | Invitrogen A21206 | 1:500 |
| 568 donkey-mouse | Invitrogen A10037 | 1:500 |
| 568 donkey-rabbit | Invitrogen A10042 | 1:500 |
| 647 donkey-mouse | Invitrogen A32787 | 1:500 |
| 647 donkey-rabbit | Invitrogen A32795 | 1:500 |

**Table S2.** **Primary and secondary antibodies used in western blot**

| Antibodies | Manufacturer and lot | Dilution ratio |
| --- | --- | --- |
| GFAP | DAKO-Z033401 | 1:1000 |
| β-actin | Immunoway YT0099 | 1:2000 |
| HRP-Goat anti rabbit | Beyotime A0208 | 1:2000 |
| HRP-Goat anti mouse | Beyotime A0216 | 1:2000 |

**Table S3.** **Primer Sequences for RT-qPCR Analysis**

| Gene name | Forward 5’-3 | Reverse 5’-3’ |
| --- | --- | --- |
| *Hmga2* | AGACCCAAAGGCAGCAAAAACA | TCTCCCATGAGAGTGGAAGCGA |
| *Igfbp3* | CACACCGAGTGACCGATTCC | GTGTCTGTGCTTTGAGACTCAT |
| *Tgfb1* | CTCCCGTGGCTTCTAGTGC | GCCTTAGTTTGGACAGGATCTG |
| *Ccnd1* | GCGTACCCTGACACCAATCTC | CTCCTCTTCGCACTTCTGCTC |
| *GAPDH* | GGTTGTCTCCTGCGACTTCA | TGGTCCAGGGTTTCTTACTCC |

**Table S4. Comparison of the transcriptional expression of *Hmga2* and other related genes in the retina with SI treatment and Hmga2-SI treatment**

| Symbol | HM-SI-14d vs SI-14d  log_2_ (FC) | HM-SI-14d vs SI-14d  FDR | HM-SI-28d vs SI-28d  log_2_ (FC) | HM-SI-28d vs SI-28d  FDR |
| --- | --- | --- | --- | --- |
| *Hmga2* | 2.43 | 2.51E-06 | 1.95 | 3.58E-05 |
| *Vimentin* | -0.35 | 0.009898178 | -0.5 | 3.24E-05 |
| *Arrestin* | 0.09 | 0.990785225 | 0.49 | 2.22E-08 |
| *Igf2bp2* | 0.5 | 0.040885837 | 0.94 | 0.000205718 |
| *Hes5* | 1.81 | 0.012294188 | 1.02 | 0.003642169 |
| *Gnas* | -0.31 | 0.111412825 | -0.32 | 0.001289692 |
